# Cell softness regulates tumorigenicity and stemness of cancer cells

**DOI:** 10.15252/embj.2020106123

**Published:** 2020-12-04

**Authors:** Jiadi Lv, Yaoping Liu, Feiran Cheng, Jiping Li, Yabo Zhou, Tianzhen Zhang, Nannan Zhou, Cong Li, Zhenfeng Wang, Longfei Ma, Mengyu Liu, Qiang Zhu, Xiaohan Liu, Ke Tang, Jingwei Ma, Huafeng Zhang, Jing Xie, Yi Fang, Haizeng Zhang, Ning Wang, Yuying Liu, Bo Huang

**Affiliations:** ^1^ Department of Immunology & National Key Laboratory of Medical Molecular Biology Institute of Basic Medical Sciences Chinese Academy of Medical Sciences (CAMS) & Peking Union Medical College Beijing China; ^2^ Institute of Microelectronics Peking University Beijing China; ^3^ Beijing Smartchip Microelectronics Technology Company Limited Beijing China; ^4^ Department of Biochemistry & Molecular Biology Tongji Medical College Huazhong University of Science & Technology Wuhan China; ^5^ National Cancer Center/Cancer Hospital CAMS Beijing China; ^6^ Deaprtment of Mechanical Science and Technology The Grainger College of Engineering University of Illinois at Urbana‐Champaign Urbana IL USA; ^7^ Clinical Immunology Center CAMS Beijing China

**Keywords:** BCL9L, metastasis, microfluidic sorting, soft tumor cells, stemness, Cancer, Cell Adhesion, Polarity & Cytoskeleton, Regenerative Medicine

## Abstract

Identifying and sorting highly tumorigenic and metastatic tumor cells from a heterogeneous cell population is a daunting challenge. Here, we show that microfluidic devices can be used to sort marker‐based heterogeneous cancer stem cells (CSC) into mechanically stiff and soft subpopulations. The isolated soft tumor cells (< 400 Pa) but not the stiff ones (> 700 Pa) can form a tumor in immunocompetent mice with 100 cells per inoculation. Notably, only the soft, but not the stiff cells, isolated from CD133^+^, ALDH^+^, or side population CSCs, are able to form a tumor with only 100 cells in NOD‐SCID or immunocompetent mice. The Wnt signaling protein BCL9L is upregulated in soft tumor cells and regulates their stemness and tumorigenicity. Clinically, BCL9L expression is correlated with a worse prognosis. Our findings suggest that the intrinsic softness is a unique marker of highly tumorigenic and metastatic tumor cells.

## Graphical abstract

Intrinsic mechanical properties and BCL9L expression in tumor cells determine their stemness and are linked to cancer progression.

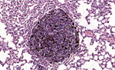

## Introduction

The notion of “cancer stem cells” or tumorigenic cells has been based on the observation that only a very small population of cells from a tumor can seed and form a tumor in severe combined immunodeficient (SCID) mice (Lapidot *et al*, 1994; Al‐Hajj *et al*, 2003; Hope *et al*, 2004; Singh *et al*, 2004; O'Brien *et al*, 2007; Quintana *et al*, 2008; Schatton *et al*, 2008). These tumorigenic cells are viewed as the source of treatment resistance and relapse of a tumor, making them a tempting therapeutic target. However, despite intensive studies, the properties of this crucial tumor cell subset remain poorly understood. Furthermore, rigorous methods are not available to isolate these cells from a tumor, because the conventional cell surface markers are unreliable and highly variable among different cancers (Hope *et al*, 2004; Dieter Sebastian *et al*, 2011). Thus, developing a method that can effectively sort and define tumorigenic cells is extremely desirable. Published reports have highlighted the importance of the mechanical properties of a living cell in cell behaviors and functions (Engler *et al*, 2006; Chowdhury *et al*, 2010; Urbanska *et al*, 2017). It is known that cells apply actomyosin‐dependent contractile forces in response to the increasing stiffness of the extracellular matrices (ECMs) (Discher *et al*, 2005; Irianto *et al*, 2016). Such endogenous contraction, in turn, can elevate cell stiffness (Wang *et al*, 1993). Moreover, to properly sense and respond to the surrounding mechanical cues, the stiffness of a cell should match that of the ECMs (Discher *et al*, 2005; Discher *et al*, 2009; Wu *et al*, 2018), suggesting that soft cells should survive in a soft stroma and stiff cells behave optimally in a stiff niche. As a support, we have demonstrated that 3D soft fibrin matrices promote H3K9 demethylation and increase Sox2 expression and self‐renewal of melanoma stem cells, whereas stiff ones exert opposite effects (Tan *et al*, 2014). In line with this notion, cells with various degrees of stiffness can co‐exist within the same tumor tissue, due to the heterogeneity of the tumor mechanical microenvironments (Plodinec *et al*, 2012; Elosegui‐Artola *et al*, 2014). Despite such understanding, direct evidence that the cellular softness functions as a basic feature for tumorigenic cells remains elusive. In this study, we develop a method to separate soft cells from stiff ones and provide evidence that these soft cells are highly tumorigenic and possess the ability to metastasize.

## Results

### Soft tumor cells are sorted by microfluidic chip

**Figure EV1:**
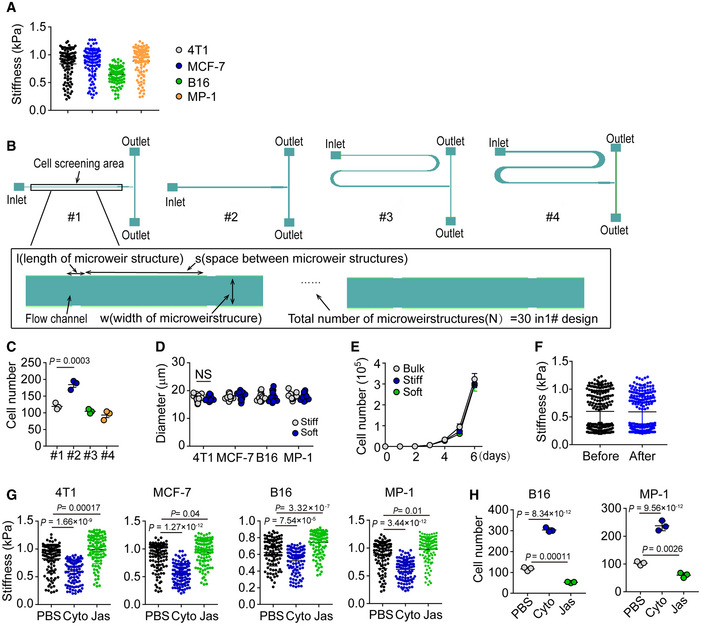
Soft tumor cells are isolated by microfluidic chip The cellular stiffness of 4T1, MCF‐7, B16F1, or MP‐1 was detected by AFM. *n* = 100.The schematic illustration of PDMS microfluidic chip fabrication for four different types. The lower is the magnification of the indicated microfluidic channel.The number of soft B16 cells isolated from four different microfluidic chips at the same flow flux of 13 μl/min for 10 min (cell density, 1 × 10^4^ cells/ml).The size of separated stiff and soft cells from 4T1, MCF‐7, B16, or MP‐1 bulk cells was measured. *n* = 10.The bulk, stiff, or soft B16 cells were cultured in 6‐well plate for the indicated time periods.The soft and stiff 4T1 cells were isolated by microfluidic chip (before) and then mixed together to be separated again by microfluidic chip (after). The stiffness of 4T1 cells from before and after re‐separation was detected by AFM. *n* = 100.The stiffness of 4T1, MCF‐7, B16, and MP‐1 cells treated with Cyto (5 μM) or Jas (50 nM) for 4 h or 12 h was measured by AFM. *n* = 100.The soft tumor cells were isolated by microfluidic chip from B16 or MP‐1 cells pre‐treated with Cyto (5 μM) or Jas (50 nM) for 4 h or 12 h. The number of soft tumor cells was counted. *n* = 3. Data information: N.S., no significant difference. Kruskal–Wallis test (G), Bonferroni test (C, D and H). The data represent mean ± SD of three independent experiments.

Indeed, atomic force microscopy (AFM) analysis showed that the stiffness of tumor cells in mouse breast cancer (4T1), human breast cancer (MCF‐7), mouse B16 melanoma, and primary human melanoma (MP‐1) was highly variable, ranging from 0.2 to 1.3 kPa. Notably, more than 60% of tumor cells had at least a stiffness of 0.7 kPa and less than 10% tumor cells had the stiffness below 0.4 kPa (Fig EV1A). Since the softness (the inverse of stiffness) renders a cell a greater degree of deformability, we thus rationally explored this as a way to separate soft tumor cells from stiff ones (Mohamed *et al*, 2009; Zhang *et al*, 2012).

**Figure 1:**
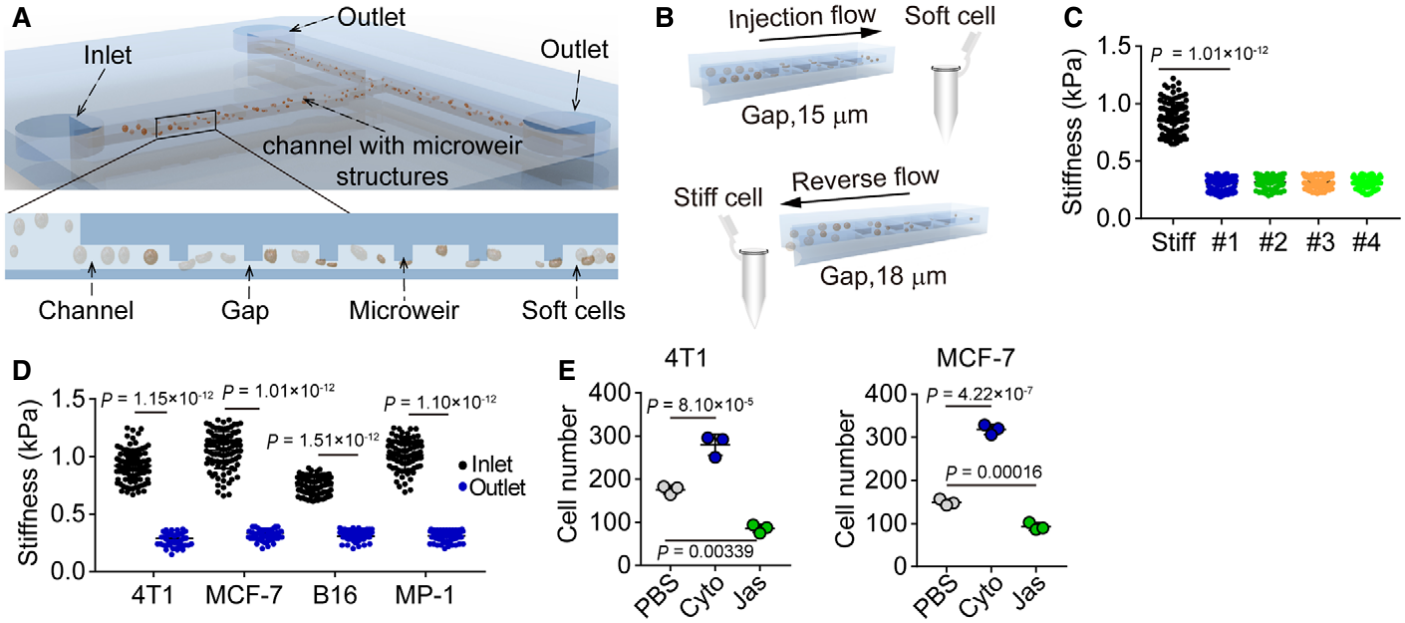
Soft tumor cells are sorted by microfluidic chip Schematic of microfluidic chip.Schematic of working principle for cell screening based on stiffness differences.The screening efficiency of four different types of chip was detected in 4T1 cells. *n* = 100.The stiffness of cells screened from the outlet or backflow inlet was measured by AFM. *n* = 100.The number of soft 4T1 or MCF‐7 cells from the outlet was calculated after treatment with or without Cyto (5 μM) or Jas (50 nM) for 4 h or 12 h, respectively. Data information: Mann–Whitney test (D), Kruskal–Wallis test (C) or one‐way ANOVA (E). The data represent mean ± SD of three independent experiments.

Here, we designed a unique microfluidic chip for label‐free cell sorting based on the cells’ physical characteristics (e.g. stiffness/deformability). The proposed microfluidic device consists of two major components, flow channels, and microweir structures (Fig 1A, upper). The gap between the microweir and main channel can act as a passive and selective barrier to isolate cells with a variety of stiffness (Fig 1A, lower). Considering that tumor cells usually have the size around 20–25 μm (Hosokawa *et al*, 2010; Hvichia *et al*, 2016), in this study, the microfluidic chips were fabricated with a 15 μm gap generation by setting the height of the microweir and flow channels at 25 and 40 μm, respectively. According to previous literatures (Mohamed *et al*, 2009; Zhang *et al*, 2012), four different types of chips (with different lengths, widths of microweir structure, spaces between microweir structures, and total numbers of microweir structures, shown in Fig EV1B and Table EV1) were designed and tested to perform the sorting. Cells (density ranging from 1 × 10^4^ to 2 × 10^4^/ml) with different degree of stiffness were injected into the chip via a syringe pump at a flow flux of 10 μl/min (Fig 1B). The cells, as they flowed out from the outlet, were collected as soft cells. In addition, we also pumped the bulk tumor cells through the microfluidic channels with a larger gap (18 μm), and inversely washed the channels. Cells then flowed out from backflow inlet and were collected as stiff cells (Fig 1B). To verify that these separated cells are authentically soft or stiff ones, we used AFM to measure the stiffness of cells. Indeed, the cells which flowed out from the outlet of each type of microtube were much softer than the cells which flowed out from the inlet (Fig 1C). Notably, the microfluidic tube #2 (260 μm distance between ridges and 11.44 mm channel length) displayed the highest sorting efficiency (Fig EV1C) and thus was used for the following experiments. Tumor cells isolated from melanoma (B16F1 and MP‐1) and breast cancer (4T1 and MCF‐7) by the microfluidic tube all displayed the soft trait (Fig 1D). We found that the separated stiff and soft cells had a similar cellular size (Fig EV1D). Also, the stiff and soft tumor cells displayed a similar growth curve in *in vitro* culture (Fig EV1E). Given that the stiffness of the isolated soft tumor cells was less than 0.4 kPa and the stiffness of the stiff cells was larger than 0.65 kPa, we defined soft cells as those with < 0.4 kPa stiffness and stiff cells as those with > 0.7 kPa stiffness in this study. In addition, we mixed the isolated soft and stiff cells for re‐separation using the microfluidic chip. We found that the distribution of soft cells before re‐separation was completely consistent with those following re‐separation (Fig EV1F), indicating that this microfluidic tube does not alter the original stiffness of the cells. F‐actin is an essential element that contributes to cellular stiffness (Wang *et al*, 1993). Cytochalasin D (Cyto), an inhibitor of actin polymerization, can decrease cell stiffness; In contrast, jasplakinolide (Jas), a natural cyclodepsipeptide that is a potent inducer of actin polymerization, can increase cell stiffness (Fig EV1G). Following the Cyto or Jas treatment of tumor cells (4T1, MCF‐7, B16, or MP‐1), the number of soft tumor cells from the outlet was increased by Cyto but decreased by Jas (Figs 1E and EV1H). Thus, a marginal population of tumor cells with the mechanical property of softness can be separated from the bulk cells using the microfluidic chip.

### Soft cells are highly tumorigenic and metastatic

**Figure 2:**
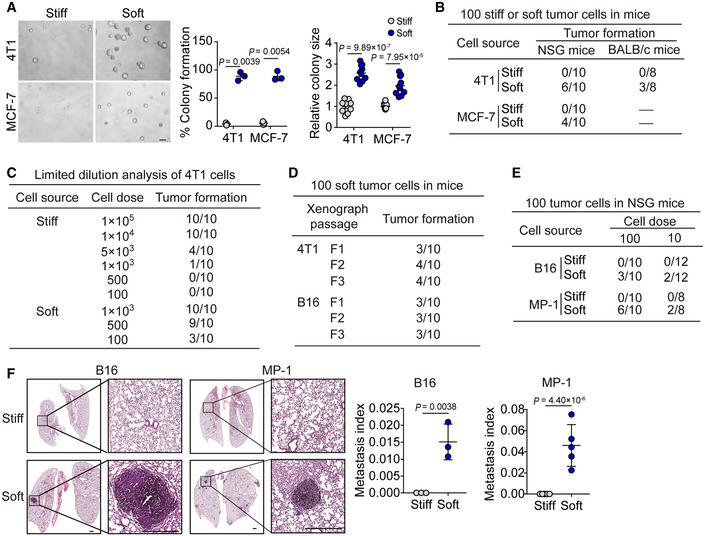
Soft tumor cells have the ability to form tumors in mice Soft or stiff 4T1 or MCF‐7 cells were isolated by the microfluidic chip, and then seeded (500 cells) in 90 Pa soft 3D fibrin gel for 3 days. The percentage of colonies formed was calculated and the colony size was recorded. Scale bar, 100 μm.The soft or stiff 4T1 or MCF‐7 cells (100 cells) were injected into the mammary fat pads of NSG or BALB/c mice. Twelve weeks later, the tumor formation was recorded. *n* = 8–10.The same as (B), except that different number of 4T1 cells were injected into the BALB/c mice. *n* = 10.The tumor‐forming capacity from primary xenografts (F1) and tumors passaged into secondary (F2) and tertiary (F3) recipients induced by injecting 100 soft 4T1 or B16 cells into the BALB/c or C57BL/6 mice. *n* = 10.Stiff or soft B16 or MP‐1 cells (100 or 10 cells) were injected into the NSG mice by tail veil. *n* = 8–12.NSG mice were intravenously injected with 100 soft or stiff B16 or MP‐1 cells. Eight weeks later, the lung metastasis was analyzed by H&E staining. The metastasis index was defined as the percentage of total metastatic nodule area to the total lung area based on the calculation from 10 slides. Scale bar, 0.5 mm. *n* = 3 (for B16) or 5 (for MP‐1) mice with metastatic tumor. Data information: Two‐tailed Paired Student’s *t*‐test (A and F). The data represent mean ± SD. *n* = 3 independent experiments in (A).

**Figure EV2:**
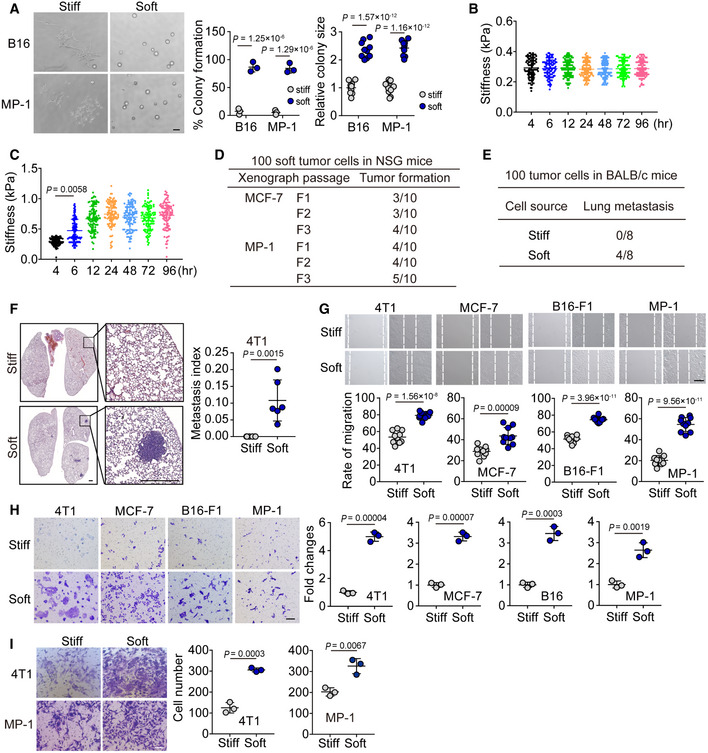
Soft tumor cells have a greater ability to migrate and invade compared with the stiff cells AThe stiff or soft B16 or MP‐1 cells were isolated by microfluidic chip, and then 500 soft or stiff cells were seeded in a 90 Pa soft 3D fibrin gel for 3 days. The percentage of colony formation was calculated, and the colony size was recorded. Scale bar, 100 μm. *n* = 3 for colony number and 10 for colony size.B, CSoft MCF‐7 cells isolated by the microfluidic chip were cultured in soft 3D fibrin gel (B) or rigid flask (C) for 4, 6, 12, 24, 48, 72, and 96 h. Stiffness of the cells was determined by AFM. *n* = 100.DThe tumor‐forming capacity from primary xenografts (F1) and tumors passaged into secondary (F2) and tertiary (F3) recipients induced by injecting 100 soft MCF‐7 or MP‐1 cells into NSG mice. *n* = 10.E, F100 stiff or soft 4T1 cells were injected into the mammary fat pads of BALB/c (E) or NSG (F) mice (*n* = 8). Eight weeks later, the lung metastasis was counted (E) and analyzed by H&E staining (F), *n* = 6 mice with metastatic tumor. Scale bar, 0.5 mm.GThe soft or stiff B16‐F1, MP‐1, 4T1, or MCF‐7 cells were grown to confluence. Then, cells were scratched and wound closure was recorded at 24 h by phase contrast microscopy. Representative images of are shown. Wound closure was calculated using ImageJ software and expressed as a percentage of the initial scratched area. Scale bar, 250 μm. *n* = 10.HThe stiff or soft 4T1, MCF‐7, B16‐F1, or MP‐1 cells were added into the hanging insert for 24 h (4T1) or 48 h (MCF‐7, B16‐F1, or MP‐1). Then, the non‐migrating cells were removed from the upper surface of the membrane, and cells that migrated through the 8 µm pore membrane were fixed and stained with 0.1% crystal violet. Scale bar, 50 μm. *n* = 3.IThe stiff or soft 4T1 or MP‐1 cells were added to the top of a matrigel invasion chamber for 24 h or 48 h. Then, the non‐invasive cells were removed from the upper surface of the membrane, and the invading cells were fixed and stained with 0.1% crystal violet. Representative pictures of invading cells and the quantification of cell invasion are shown. Scale bar, 50 μm. *n* = 3. Data information: Two‐tailed paired Student’s *t*‐test (A and F–I), Kruskal–Wallis test (C). The data represent mean ± SD. Source data are available online for this figure.

Next, we investigated the biological property of the soft tumor cells. Our previous studies had demonstrated that tumorigenic cells rather than differentiated tumor cells are selected and grow in 90 Pa soft 3D fibrin gels (Liu *et al*, 2012; Liu *et al*, 2018); and these selected cells are physically much softer than the differentiated counterparts and can be represented by CD133^hi^ melanoma cells or ALDH^+^ breast cancer cells *in vivo* (Liu *et al*, 2018). These findings prompted us to hypothesize that mechanical softness might be a common feature for tumorigenic cells. To test this hypothesis, we seeded the soft or stiff tumor cells following separation by a microfluidic channel (B16, MP‐1, 4T1, MCF‐7) into the 90 Pa soft 3D fibrin gels. We found that greater than 95% of soft tumor cells could form colonies, while stiff tumor cells only formed few colonies with a much smaller size (Figs 2A and EV2A). Meanwhile, we found that the softness of the isolated soft MCF‐7 cells could be kept in soft fibrin gels, while, the soft cells, if seeded in rigid culture plates for 4 h, started to become stiff and reached the stiff peak 12 h later. Next, we injected 100 separated soft or stiff cells (4T1 and MCF‐7) into the mammary fat pads of NOD/SCID IL‐2Rγ‐null (NSG) mice. Twelve weeks later, the 100 soft cells could form a tumor *in situ* with a relatively high frequency (6/10 for 4T1, 4/10 for MCF‐7), while the 100 stiff cells injected did not form a tumor (Fig 2B). Moreover, 100 soft 4T1 cells could even form a tumor in immunocompetent mice (wild‐type BALB/c) with a rate of tumor formation (3/8), suggesting that the soft tumor cells have a highly tumorigenic ability. In addition, when we performed limiting dilution experiments to quantify the frequency of tumors for each condition of soft or stiff cell inoculation (O'Brien *et al*, 2007), we found that the highest frequency of tumor formation occurred in the soft cell group (Fig 2C). Moreover, by conducting serial transplants with 100 soft tumor cells, we found that the tumor size and appearance of the first implantation was similar to the second and third generation of passaged tumors in the mice (Figs 2D and EV2D). A cardinal feature of malignant melanoma is its inclination to metastasize to the lungs. Eight weeks following an intravenous injection of 100 soft or stiff cells separated from B16‐F1 or MP‐1 in NSG mice, metastatic tumors in the lungs were visible from the soft cell group. Interestingly, even as few as 10 soft cells could generate metastatic tumors (2/12 for B16 or 2/8 for MP‐1), but no metastatic tumors were detected in the lungs following the injection of 100 stiff cells (Fig 2E and F). Then, we used the 4T1 cell lung metastasis model to further validate this result. The soft or stiff 4T1 cells were injected into the mammary fat pads of WT BALB/c mice. Eight weeks later, the mice were sacrificed for H&E staining of the lungs, showing metastatic tumors (4/8) in the soft cell group (Fig EV2E and F). However, no lung metastatic tumors (0/8) were observed in the stiff cell group. In line with these *in vivo* results, the soft tumor cells displayed greater ability to migrate and invade *in vitro*, compared with the stiff cells (Fig EV2G–I). Together, these data suggest that soft tumor cells are highly tumorigenic in their ability to form a tumor at both primary and metastatic sites.

### Softness is a physical marker for tumorigenic cells

**Figure 3:**
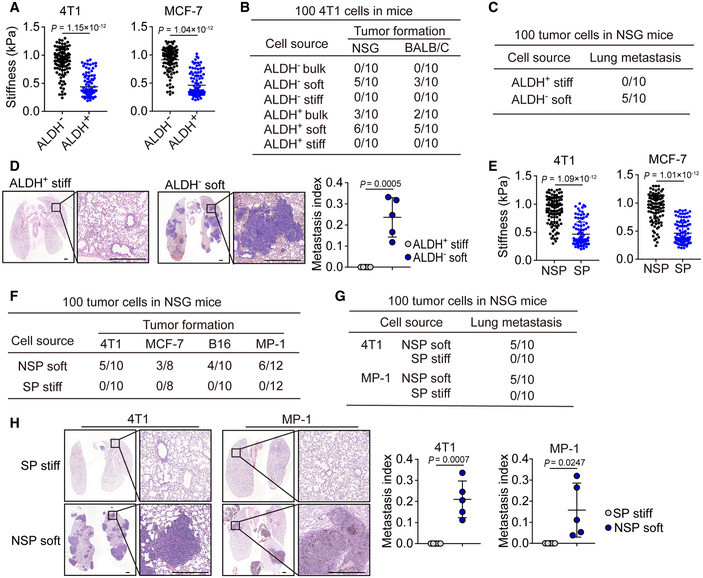
Tumorigenic cells are characterized by mechanical softness AThe cellular stiffness of ALDH^−^ and ALDH^+^ 4T1 or MCF‐7 cells sorted by flow cytometry were determined by AFM. *n* = 100.BThe stiff or soft cells were isolated from ALDH^−^ or ALDH^+^ 4T1 cells by microfluidic chip. Then, these cells were injected into the mammary fat pads of NSG or BALB/c mice (100 cells/mouse). Twelve weeks later, the tumor formation was recorded. *n* = 10.C, DThe 100 stiff ALDH^+^ or soft ALDH^−^ 4T1 cells were injected into the mammary fat pads of NSG mice. Eight weeks later, the lung sections were H&E stained. The lung metastatic mice were counted (C), and the metastasis index was calculated (D). Scale bar, 0.5 mm. *n* = 5 mice with metastatic tumor.EThe side population (SP) or non‐SP (NSP) from 4T1 or MCF‐7 cells were sorted by flow cytometry, and then, the cellular stiffness of those cells was measured by AFM. *n* = 100.FThe 100 soft NSP or stiff SP‐ 4T1, MCF‐7, B16, or MP‐1 cells were injected into the mammary fat pads (4T1 and MCF‐7) or subcutaneous tissue (B16 and MP‐1) of NSG mice. The tumor formation in the lungs was recorded.G, HThe 100 soft NSP or stiff SP‐ 4T1 or MP‐1 cells were injected into the mammary fat pads (for 4T1) of NSG mice or by tail vein into NSG mice (for MP‐1). The tumor formation in the lungs was recorded (G). Six to eight weeks later, mice were sacrificed and the lung sections were H&E stained. The metastatic micronodules in the lung were counted, and the metastasis index was calculated (H). Scale bar, 0.5 mm. *n* = 5 mice with metastatic tumor. Data information: Mann–Whitney test (A and E), or two‐tailed paired Student’s *t*‐test (D and H). The data represent mean ± SD.

**Figure EV3:**
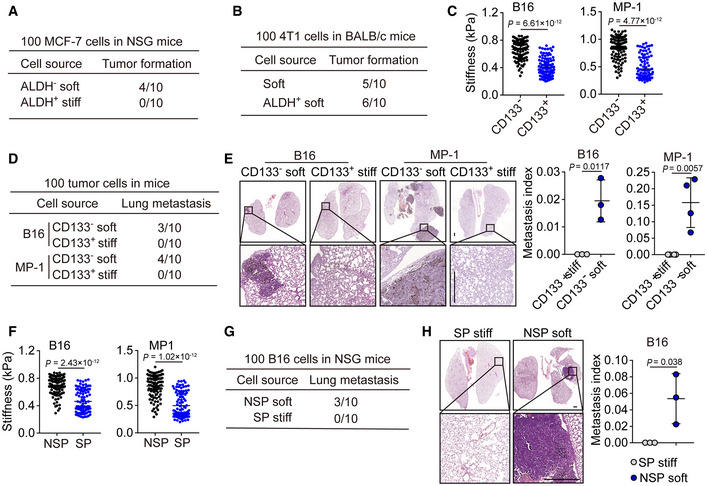
Mechanical softness is the key characteristic of tumorigenic cells AThe ALDH^−^, or ALDH^+^ stiff, or soft MCF‐7 cells were injected into the mammary fat pads of NSG mice (100 cells/mouse). The tumor formation was recorded. *n* = 10.BThe same as (A), except that soft 4T1 or ALDH^+^ soft 4T1 cells were injected into BALB/c mice. *n* = 10.CThe stiffness of CD133^−^ or CD133^+^ B16 or MP‐1 cells was measured by AFM. *n* = 100.D, EThe 100 stiff CD133^+^ or soft CD133^−^ B16 or MP‐1 cells were injected into the NSG mice by tail vein injection. Six weeks later, mice were sacrificed and the lung sections were H&E stained. The mice with lung metastasis were recorded (D). The metastatic micronodules in the lung were counted, and the metastasis index was calculated (E). Scale bar, 0.5 mm. *n* = 3 (B16) or 4 (MP‐1) mice with metastatic tumor.FThe stiffness of the side population (SP) or non‐SP (NSP) B16 or MP‐1 cells were detected by AFM. *n* = 100.G, HThe 100 soft NSP or stiff SP‐B16 cells were injected i.v. into NSG mice for 6 weeks. The tumor formation in the lungs was recorded (G). The lung sections perform H&E stained. The metastatic micronodules in the lung were counted, and the metastasis index was calculated (H). Scale bar, 0.5 mm. *n* = 3 mice with metastatic tumor. Data information: Mann–Whitney test (C and F), Paired Student’s *t*‐test (E and H). The data represent mean ± SD.

Next, we asked whether this mechanical softness could function as a useful marker for tumorigenic cells. Chemical molecules on the cell surface or in the cytosol, such as the enzyme ALDH1, have been widely used to denote cancer stem cells despite their unreliability and low specificity (Ginestier *et al*, 2007; Douville *et al*, 2009). When we used softness to analyze ALDH1^+^ and ALDH1^−^ breast cancer cells (4T1 and MCF‐7), we found that around 65% ALDH1^+^ cells were soft and around 6% ALDH1^−^ cells were soft (Fig 3A). Intriguingly, inoculation of 100 soft either ALDH1^+^ or ALDH1^−^ tumor cells were able to form a tumor in either NSG or WT mice; however, inoculation of 100 stiff ALDH1^+^ or ALDH1^−^ tumor cells could not form a tumor, even in NSG mice (Figs 3B and EV3A). In addition, the inoculation of 100 unseparated ALDH1^+^ tumor cells displayed half the tumorigenic capability of the sorted soft ones; however, these sorted soft ones had the similar tumorigenic capability as the sorted ALDH1^+^ soft tumor cells (Figs 3B and EV3B). Consistently, only soft ALDH1^−^ (100) but not stiff ALDH1^+^ ones were able to form lung metastatic micronodules (Fig 3C and D). Besides 4T1 and MCF‐7, similar results were also obtained from B16 and MP‐1 melanoma cells. We found that ~ 60% CD133^+^ and ~ 4% CD133^−^ cells were soft (Fig EV3C), and 100 CD133^−^ soft cells had the ability to form a subcutaneous tumor and lung metastasis, but this was not observed with the 100 CD133^+^ stiff cells (Fig EV3D and E). Thus, surface marker‐defined cancer stem cells may contain both the soft and stiff subpopulations and only the soft subset has a tumorigenic capability. To further validate this conclusion, we used a comparable approach to isolate a stem cell‐like side population (SP) from tumor cells, based on the efflux of Hoechst 33342 (Golebiewska *et al*, 2011). Intriguingly, among SP cells, around 50–60% cells displayed a soft property (Figs 3E and EV3F). Also, the isolated SP and non‐SP (NSP) cells (4T1 or MCF‐7) were further separated into stiff and soft subpopulations using microfluidic devices. As expected, the injection of 100 soft NSP tumor cells into the mammary fat pads of NSG mice led to visible tumor formation within 12 weeks at a ~ 50% frequency, but the injection of stiff SP^+^ cells did not cause detectable tumors (Fig 3F). Similar results were obtained from the stiff SP or soft NSP B16 or MP‐1 cells (Fig 3F). In addition, metastatic micronodules in the lungs were found by H&E staining from the soft NSP group but not from the stiff SP group on day 80 (Figs 3G and H, and EV3FG and H). Thus, SP cells also contain soft subpopulation and employ them to grow a tumor at the primary and metastatic sites. Together, these results suggest that intrinsic softness may be a suitable characteristic to mark tumorigenic cells.

### Soft tumor cells upregulated Wnt‐BCL9L pathway for stemness development

**Figure 4:**
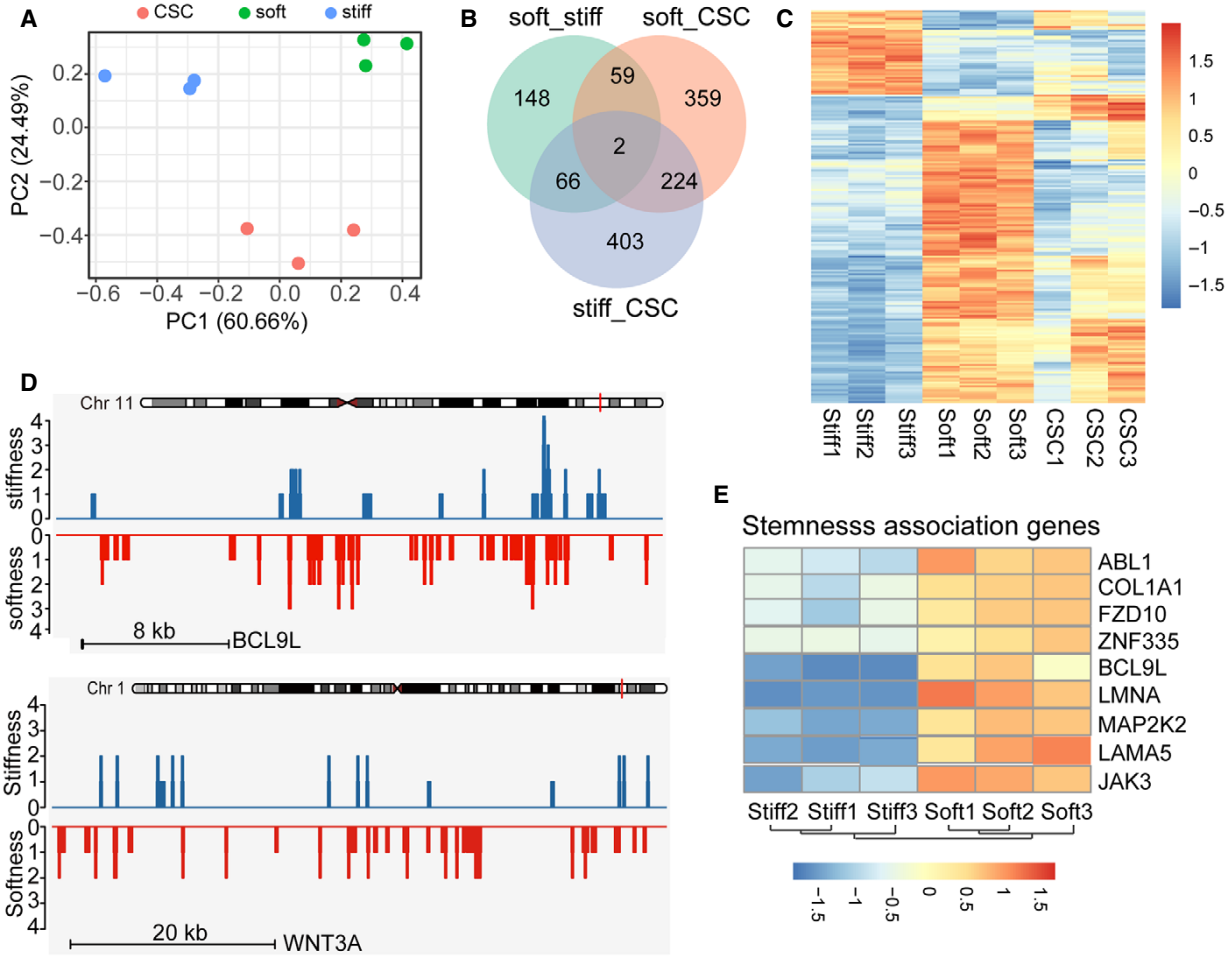
Stemness associated genes were identified from soft tumor cells PCA plot showing the clustering of stiff, soft cells, and cancer stem cells (CSC). The variance is the smallest between stiff and soft on PC2.Venn diagram comparing the hits from stiff, soft, or CSC MCF‐7 cells.Heatmap of differentially mRNA‐seq expressed genes in stiff, soft, or ALDH^+^ (CSC) MCF‐7 cells. The unit for the color scale was z‐score of log2 expression data shown.ATAC–seq tracks of BCL9L and WNT3A in stiff or soft cells.Heatmap of stemness association genes expression determined by mRNA sequencing comparing stiff and soft MCF‐7 cells. *n* = 3. The unit for the color scale was *z*‐score of log_2_ expression data shown.

**Figure EV4:**
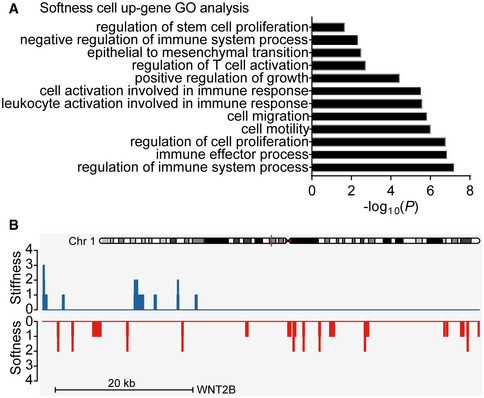
RNA‐seq and ATAC analysis of stiff, soft, and ALDH^+^ cells (CSC) The GO analysis of softness cells’ upregulated genes was performed.ATAC‐seq tracks of WNT2B in stiff or soft cells.

Furthermore, we examined the biological difference between soft and stiff cells at the genetic levels in order to better understand the merit of softness as a marker for tumorigenic cells. To this end, we began by sequencing the entire genome of the soft tumorigenic, ALDH^+^, and stiff differentiated cells, respectively. We did not find a difference at the DNA levels among the three groups. Subsequently, we assessed the gene expression pattern by mRNA sequencing. Using principal component analysis (PCA), we found that the difference between the soft and stiff groups was much larger than that between the CSC and stiff groups, while the internal variation in the soft group was smaller than that in the CSC group (Fig 4A). Meanwhile, the differentially expressed genes among the soft, stiff, and CSC cells were also shown by Venn diagram (Fig 4B). Gene ontology analysis identified these differentially expressed gene families to be associated with stem cell proliferation, cell migration, and immune system processes, et al. (Fig EV4A). Further, the top 2,000 genes differentially expressed among soft, stiff, and CSC cells were listed to produce a graphical heat map (Fig 4C), presenting a more tightly clustered genes between the soft and stiff groups relative to those between the CSC and stiff groups, suggesting that softness is better than molecular markers at representing the inherent characteristic of tumorigenic cells. Using a comparable approach, we also performed a chromatin accessibility analysis through ATAC‐seq, which revealed many differentially accessible peaks in soft and stiff tumor cells. Of note, BCL9L, WNT2B, and WNT3A were among the most differentially expressed genes and were also more prominent at the chromatin‐opening state in soft tumor cells (Figs 4D and EV4B). In addition, the analysis of stemness‐associated genes derived from RNA sequencing also indicated BCL9L as a strong candidate (Fig 4E). Coincidently, Wnt signaling is critical in the regulation of stem cell pluripotency and self‐renewal; however, BCL9/BCL9L act additional transcriptional co‐activators and form part of the Wnt enhanceosome (van Tienen *et al*, 2017). Thus, we focused on BCL9L (B‐cell CLL/lymphoma 9‐like), a homologue of BCL9 with functional redundancy.

### BCL9L is a stemness marker for soft tumor cells

**Figure 5:**
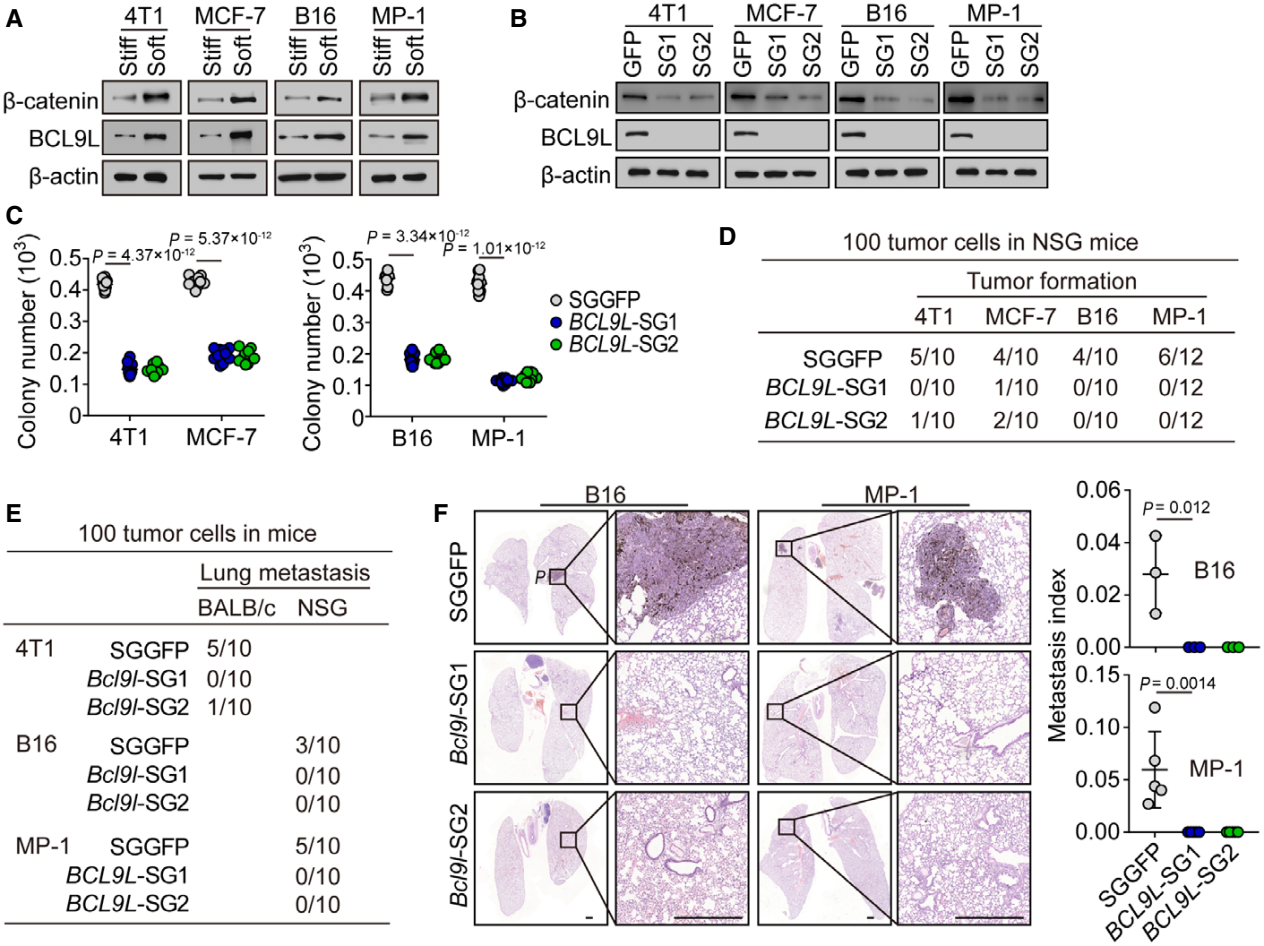
BCL9L is the key mediator to regulate the stemness and tumorigenicity of soft tumor cells AThe expression of BCL9L or β‐catenin in stiff or soft 4T1, MCF‐7, B16, or MP‐1 cells was determined by Western blot.BThe expression of β‐catenin and BCL9L from SGGFP, *BCL9L*‐SGs‐4T1, MCF‐7, B16, or MP‐1 cells were determined by Western blot.C500 soft SGGFP or *BCL9L*‐SGs‐ 4T1, MCF‐7, B16‐F1, or MP‐1 cells were seeded in a 90 Pa soft 3D fibrin gel for 6 days. The number of formed colonies was counted. *n* = 10.DThe soft SGGFP or *BCL9L*‐SGs‐ 4T1, MCF‐7, B16, or MP‐1 cells (100 cells) were injected into the mammary fat pads (4T1 and MCF‐7) or subcutaneous tissue (B16 and MP‐1) of NSG mice. Tumor formation was recorded. *n* = 10 or 12 mice as indicated.E, FThe 100 soft SGGFP or *BCL9L*‐SGs‐ 4T1, B16, or MP‐1 cells were injected into the mammary fat pads (4T1) of BALB/c mice or tail vein (B16 and MP‐1) of NSG mice. The tumor formation was recorded (E). Six to eight weeks later, the lung sections were H&E stained. The metastatic micronodules in the lung were counted, and the metastasis index was calculated (F). Scale bar, 0.5 mm. *n* = 3 (for B16‐F1) or 5 (for MP‐1) mice with metastatic tumor. Data information: Bonferroni test (C and F). The data represent mean ± SD. *n* = 3 independent experiments in (A and B). Source data are available online for this figure.

**Figure EV5:**
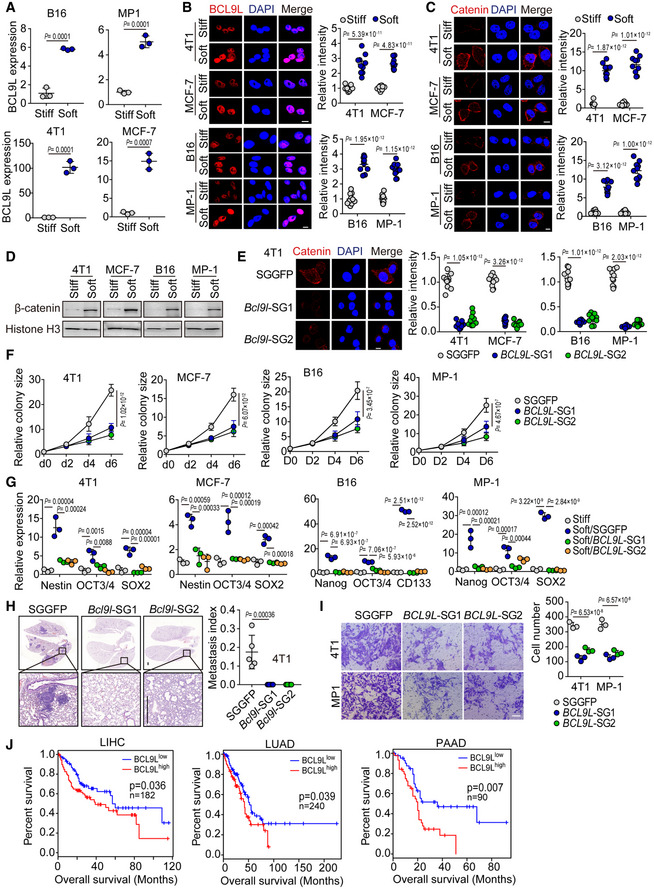
BCL9L participates in regulating the stemness of soft tumor cells AThe mRNA expression of BCL9L in stiff or soft B16, MP‐1, 4T1, or MCF‐7 cells was determined by real‐time PCR. *n* = 3.B, CThe stiff or soft B16, MP‐1, 4T1, or MCF‐7 cells were stained with anti‐BCL9L (B) or anti‐β‐catenin (C) and DAPI and observed under confocal microscope. The relative intensity was quantified. Scale bar, 10 μm. *n* = 10.DThe expression of nuclear β‐catenin in stiff or soft 4T1, MCF‐7, B16, or MP‐1 cells was determined by Western blot.ESGGFP, *BCL9L*‐SGs‐ 4T1, MCF‐7, B16, or MP‐1 cells were stained with anti‐β‐catenin antibody and observed under confocal microscope. The relative intensity was quantified. Scale bar, 10 μm. *n* = 10.F500 soft SGGFP or *BCL9L*‐SGs‐4T1, MCF‐7, B16, or MP‐1 cells were seeded in 90 Pa soft 3D fibrin gel for the indicated time periods. The colony size was measured. The colony size in D0 (day 0) was set to 1. *n* = 3.GThe mRNA expression of Nestin, Nanog, OCT3/4, CD133, or SOX2 in stiff or soft SGGFP or *BCL9L*‐SGs‐ B16, MP‐1, 4T1, or MCF‐7 cells was detected by real‐time PCR. *n* = 3.HThe 100 soft SGGFP or *BCL9L*‐SGs‐4T1 cells were injected into the mammary fat pads of NSG mice for 8 weeks. Then, mice were sacrificed and the lung sections were performed H&E stained. The metastasis index was calculated. *n* = 5 mice with metastatic tumor. Scale bar, 0.5 mm.IThe Transwell invasion assay from soft SGGFP‐ or *BCL9L*‐SGs‐ 4T1 or MP‐1 cells. The number of invading cells was calculated from 3 fields. *n* = 3. Scale bar, 50 μm.JOverall survival compared with the BCL9L level in patients with liver (LIHC, *n* = 182), lung (LUAD, *n* = 240), or pancreatic (PAAD, *n* = 90) cancer. Data information: Paired Student’s *t*‐test (A–C), Bonferroni test (E–I) or Log‐rank survival analysis (J). The data represent mean ± SD. Source data are available online for this figure.

Indeed, we confirmed that the expression of BCL9L was remarkably upregulated in soft breast (4T1 and MCF‐7) and melanoma (B16 and MP‐1) tumor cells, as evidenced by qPCR, Western blot, and immunostaining (Figs 5A, and EV5A and B). In line with this upregulation of BCL9L, the expression of the total β‐catenin and its nuclear form was also enhanced in the soft tumor cells and an enhanced nuclear translocation was observed (Figs 5A, and EV5C and D). Notably, the knockout of BCL9L by CRISPR/Cas9 resulted in the downregulation of β‐catenin expression at both the whole cell and nuclear levels in the soft tumor cells (Figs 5B and EV5E). Also, this BCL9L knockout decreased colony number and reduced the colony size of tumor cells in the soft 3D fibrin gels (Figs 5C and EV5F). In addition, we found that the expression of stemness genes was upregulated in the soft tumor cells (Nestin, OCT3/4 and SOX2 for 4T1/MCF‐7; Nanog, OCT3/4, SOX2, and CD133 for B16F1/MP‐1), while the BCL9L knockout reversed this effect (Fig EV5G). To further validate these *in vitro* results *in vivo*, we inoculated mice with 100 soft SGGFP‐ or *BCL9L*‐SGs‐tumor cells. Twelve weeks later, we found that the soft SGGFP‐tumor cells formed a tumor in the mice; in contrast, no tumors were formed in the BCL9L knockout group (Fig 5D). Also, the BCL9L knockout abrogated the lung metastasis of soft tumor cells (Figs 5E and F, and EV5H). In line with this *in vivo* result, BCL9L knockout also inhibited soft tumor cell invasion *in vitro* (Fig EV5I). Together, these results suggest that BCL9L is an intrinsic molecular component in soft cells utilized to maintain their stemness.

### Soft tumor cells highly expressed BCL9L in patients

**Figure 6:**
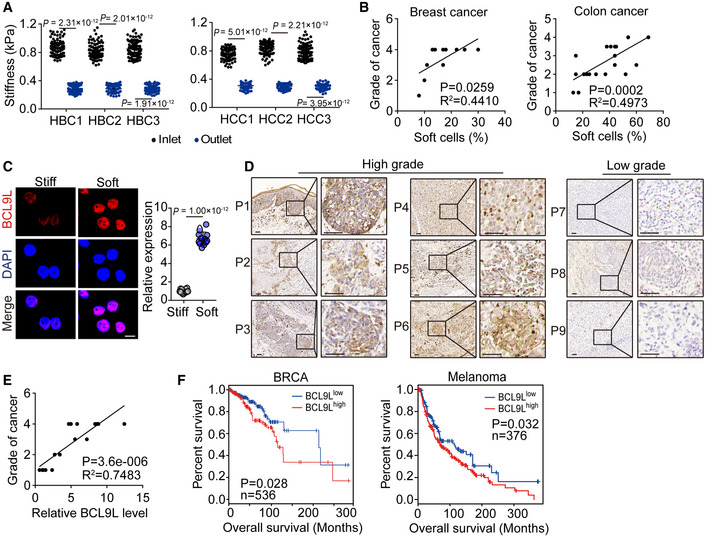
BCL9L highly expressed in soft tumor cells from cancer patients The stiffness of primary breast (HBC, *n* = 3) and colon (HCC, *n* = 3) cancer cells screened from the outlet or backflow inlet was measured by AFM. *n* = 100 from each patient.The correlation between the percentage of soft primary tumor cells from patients with breast (*n* = 11) or colon (*n* = 23) cancer and the grade of breast or colon cancer, respectively.The stiff and soft tumor cells were isolated from 3 patients with breast cancer and stained with anti‐BCL9L antibody. The expression of BCL9L was observed under confocal microscope. Scale bar, 10 μm.The tissue sections from 6 melanoma patients with high grades of cancer and 3 melanoma patients with low grade of malignancy were immunohistochemical staining with anti‐BCL9L antibody. Scale bar, 50 μm.The correlation between the expression of BCL9L from 18 patients with melanoma and the grade of melanoma.Overall survival compared with the BCL9L level in patients with breast cancer (BRCA, *n* = 536) or melanoma (*n* = 376). Data information: Mann–Whitney test (A), paired Student’s *t*‐test (C), Pearson’s correlation test (B and E) or Log‐rank survival analysis (F). The data represent mean ± SD.

Finally, we sought to validate our findings in clinical patient samples. Using microfluidic devices, we separated soft tumor cells from the breast or colon cancer tissues of patients. We found that the soft primary breast or colon cancer cells displayed a cellular stiffness of around 0.3 kPa, while their separated stiff counterparts were over 0.7 kPa (Fig 6A). Moreover, the percentage of soft primary tumor cells isolated from patients with colon (*n* = 23) or breast (*n* = 11) cancer was strongly correlated with the tumor pathological grade (Fig 6B). Then, we detected the expression of BCL9L in soft and stiff tumor cells isolated from patients with breast cancer. We found that BCL9L levels were much higher in soft tumor cells compared with stiff ones (Fig 6C). Further screening of BCL9L in 16 patients with melanoma showed that BCL9L was highly expressed in melanoma tissues and tumor BCL9L levels was positively correlated with the stage of melanoma (Fig 6D and E). Consistently, the high expression of BCL9L in tumor tissues was correlated with a poorer prognosis of patients with melanoma, breast cancer, and other cancer types such as pancreatic cancer, lung cancer, and liver cancer (Figs 6F and EV5J). These data suggested that mechanical softness might be a potential prognostic indicator for patients with cancer.

## Discussion

The CSC theory remains controversial regarding their origin, frequency, phenotype, and function (Clevers, 2011; Kaiser, 2015; Wang *et al*, 2015). Although current CSC markers enable the development of clinical diagnostics and CSC‐based therapies (Keysar & Jimeno, 2010; Medema, 2013), such markers can also be detected, more or less, on normal stem cells, differentiated cancer cells, or even normal tissues (Ginestier *et al*, 2007; Gregorieff *et al*, 2015). Furthermore, CSC markers are very unstable. For instance, both CD133^+^ and CD133^−^ tumor cells have been identified as CSCs (Singh *et al*, 2004; Beier *et al*, 2007; Shmelkov *et al*, 2008). Also, both CD34^+^CD38^−^ and CD34^+^CD38^+^ AML cells have displayed tumorigenic activity (Taussig *et al*, 2008; Taussig *et al*, 2010). Unlike the variability of biochemical molecules, the physical trait of a cell is more stable. In this study, we provide evidence that cellular softness can act as a universal marker to define CSCs.

Softness confers cells with the ability to deform. Unlike previously reported microfluidic chips, used to isolate soft cells (Mohamed *et al*, 2009; Guo *et al*, 2012), we have developed a chip with single‐stream features, such as a longer channel in a limited area, efficient collection of cells by forward and reverse flows, and convenient single‐cell observation by microscopy. The use of a silicon mold for device fabrication ensures process accuracy and the convenience for producing multiple duplicates of polydimethylsiloxane microfluidic chips, which is important for good experimental control. Using this device, we isolated both soft and stiff tumor cells. Notably, only the isolated soft tumor cells were able to form colonies in soft 3D fibrin gels but the stiff ones could not form colonies. Moreover, conventional marker‐based CSCs, such as CD133^+^, ALDH1^+^, or SP^+^ CSCs, isolated from melanoma or breast cancer cells, contain both soft and stiff subsets; and only the soft subpopulation has the ability to form a tumor. In addition to tumor formation, an important feature for CSCs lies in the ability to metastasize to distant organ(s). Consistently, the soft tumor cells display the capacity of invasion and metastasis; however, the stiff ones demonstrate poor capacity for metastasis.

Published reports have shown a positive association between increased tissue stiffness and aggressive cancer behavior, prompting to propose a model of cancer progression that depends on static or dynamic tumor tissue stiffening (Paszek *et al*, 2005; Wei *et al*, 2015; Ondeck *et al*, 2019). However, there has been no evidence to support the notion that the cells coming out of the stiffened tumor stroma are tumorigenic and metastatic cancer cells. It has also been unclear whether the tumorigenic cells are stiff cells or soft cells. Despite the overall stiffness, local microenvironments for tumor stiffness are highly heterogeneous (Plodinec *et al*, 2012). Increased tissue stiffness may be attributed to more extracellular matrices, which are likely to limit blood vessel distribution and lead to tumor hypoxia, a common phenomenon in tumor microenvironments. It is known that hypoxia in primary tumors is associated with an increased metastasis and a worse prognosis in patients with cancer (Erler *et al*, 2006; Gilkes *et al*, 2014; Rankin & Giaccia, 2016). Recently, we demonstrated that hypoxia promote human breast tumor‐repopulating cell development (Tang *et al*, 2019). Of note, hypoxic areas may be very soft due to local tissue necrosis and matrix degradation. Thus, increased tissue stiffness may result in more soft tumor cells at the hypoxic sites, favoring an aggressive cancer behavior. Consistently, studies by Superfine *et al* showed that cancer cells with the highest migratory and invasive potential are five times less stiff than cells with the lowest migration and invasion potential (Swaminathan *et al*, 2011). Our results in this study strongly suggest that only the intrinsically soft tumor cells are able to be highly tumorigenic and metastatic in animal models. Our findings are consistent with the reports that metastatic tumor cells are much softer that non‐metastatic cancer cells (Guck *et al*, 2005; Cross *et al*, 2007; Plodinec *et al*, 2012; Xu *et al*, 2012). Our soft tumor cell data are also consistent with the finding that weakly adherent tumor cells are more migratory and metastatic (Hope *et al*, 2004). In addition to hypoxia, it is possible that local tumor tissue stiffening may promote the epithelial–mesenchymal transition and thus the stiffening of tumors cells might facilitate a subpopulation of tumor cells to differentiate and to migrate out of the tumor stroma. However, it is only those soft undifferentiated tumor cells that also come out of the stroma which are able to metastasize and proliferate at the secondary sites, as we have proposed previously (Tan *et al*, 2014). Nevertheless, whether the soft tumor cells or the stiff tumor cells convey metastatic and tumorigenic potential in human patients needs to be examined carefully in the future.

CSC theory implicates the use of CSC markers to predict patient prognosis. However, existing evidence is conflicting. Some studies show that CSC markers are correlated with a poor prognosis and overall survival (Ginestier *et al*, 2007; Iinuma *et al*, 2011; Stavropoulou *et al*, 2016), but some studies indicate that CSC markers like CD133 and ALDH1 have no correlation with prognosis (Lugli *et al*, 2010; Wakamatsu *et al*, 2012; Kapucuoğlu *et al*, 2015; Miller *et al*, 2017). This might be due to the notion that marker‐based CSCs contain both soft and stiff tumor cells. Thus, the use of cellular softness may be a potential physical marker used to predict cancer patient prognosis. An important finding in this study is that BCL9L is identified as a biological marker for soft tumor cells to distinguish them from their stiff counterparts. Wnt/β‐catenin signaling plays a crucial role in the regulation of the pluripotency and self‐renewal of stem cells. To exert its function, β‐catenin enters the nucleus, where β‐catenin triggers Wnt‐mediated transcription in association with transcription factors TCF/Lef. However, BCL9L acts as an additional transcriptional co‐activator and form part of the Wnt enhanceosome. BCL9L is upregulated in soft tumor cells but very weakly expressed in stiff tumor cells. More importantly, in our small cohort study, we indeed observed that BCL9L expression is related to the prognosis of patients with cancer. Based on these analyses, we propose that BCL9L might function as a useful marker to predict cancer patient prognosis.

## Materials and Methods

### Animals and cell lines

Six‐week‐old female C57BL/6, BALB/c, and NOD/SCID IL‐2Rγ‐null mice (NSG) were purchased from the Center of Medical Experimental Animals of the Chinese Academy of Medical Science (Beijing, China). These animals were maintained in the Animal Facilities of the Chinese Academy of Medical Science under pathogen‐free conditions. All studies involving mice were approved by the Animal Care and Use Committee of the Chinese Academy of Medical Science. Murine B16 melanoma and 4T1 breast cancer, and human MCF‐7 breast cancer cell lines were purchased from the China Center for Type Culture Collection (Beijing, China). Primary tumor cells isolated from human melanoma (MP‐1) and human colorectal cancer (HCC‐5 and HCC‐6) tissue were grown in RPMI1640 medium (Gibco, USA). B16 and MCF‐7 cells were cultured in DMEM (Gibco, USA), and 4T1 was grown in RPMI1640 medium. All media was supplemented with 10% fetal bovine serum (FBS) (Gibco, USA) and 2 mM l‐glutamine (Gibco, USA). All cells were grown at 37°C in a 5% CO_2_ incubator.

### Human samples

Colon or breast cancer tissues were obtained from patients at the National Cancer Center/Cancer Hospital. Ethical permission was granted by the Clinical Trial Ethics Committee of National Cancer Center/Cancer Hospital. The paraffin embedding tumor tissues of patients with melanoma were obtained from the Department of Pathology, Cancer Hospital of Yunnan Province. Ethical review was granted by the Institutional Ethics Committee of Cancer Hospital of Yunnan Province. The clinical features of the patients are listed in Tables [Link], [Link], [Link].

### Reagents, materials, and equipment

Cytochalasin D (CD, Cat. PHZ1063) and Jasplakinolide (Jas, Cat. J4580) were purchased from Invivogen and Sigma, respectively. Silicon wafers (single crystal, n‐type doping) were purchased from No.46 Research Institute of China Electronics Technology Corporation (CETC46, Tianjin, China). RZJ‐304 photoresist used in the lithography was from Ruihong Comp. (Shanghai, China). Sylgard 184 silicone elastomer (polydimethylsiloxane, PDMS), including base and curing agent, was purchased from Dow Corning Corp. (Midland, USA). The corona plasma treater (BD‐20AC) for PDMS/glass bonding was from ETP (Electro‐Technic Products, Chicago, USA). The syringe pump (Fusion 200) used in this work was purchased from Chemyx Inc. (Stafford, USA). The plastic tube was custom‐made by Nantong Yinuo Precision Plastic Pipe Co. LTD. (Nantong, Jiangsu, China). The syringes were purchased from Shanghai Gaoge Industrial and Trading Co. LTD. (Shanghai, China).

### Fabrication of microfluidic chips

Fabrication of the PDMS soft‐lithography mold started with a 4‐inch silicon wafer. First, 3,000 Å SiO_2_ was formed by a thermal oxidation. Then, the first lithography step was conducted to define the flow channel and microweir structure followed by selective removal of the exposed SiO_2_ by BHF (buffered hydrofluoric acid) bath. Next, the second lithography and RIE (reacting ion etching) of SiO_2_ were successively conducted, as an ingenious design for elimination of alignment errors from two lithography steps. Afterwards, a first DRIE (deep reactive ion etching) was conducted with the patterned photoresist (from the second lithography) as the etching mask to form the initial depth (flow channel height minuses microweir height). After stripping the photoresist, the second DRIE (etching depth equaling to the microweir height) was applied directly to form the two‐depth silicon structure with the pre‐defined SiO_2_ as the etching mask. The final silicon mold was obtained after removing SiO_2_ in a BHF bath. Next, PDMS pre‐polymer was prepared by mixing the base and the curing agent with a weight ratio of 10:1. The mixture was degassed for 20 min in a vacuum desiccator. Then, the pre‐polymer was poured onto the surface pre‐treated (release agent coating) silicon mold and cured in an oven at 70°C for 60 min. After released from the mold, the PDMS were punched through to function as inlet/out and then bonded with a glass slide by an O_2_ plasma treatment of 10 s. In this study, chips with four different designs, including different lengths, widths of microweir structure, spaces between microweir structures, and total numbers of microweir structures were designed and investigated.

### Cell screening through the microfluidic chips

The microfluidic chip was connected to a syringe pump through plastic tubes for sample loading. The chip was first rinsed with 75% ethanol and 1% (*w*/*v*) bovine serum albumin (BSA) sequentially. Then, the cell suspension (density ranging from 1 × 10^4^ to 2 × 10^4^/ml) was injected into the chip through the inlet, driven by a syringe pump with a flow rate at 10 μl/min. After the cell solution loading, the cells getting to the outlet were collected as soft cells. For the screening of stiff cells, the residual cells in the Chip (cells that did not pass through the microweir structures) were driven back into the inlet by a reverse flow and collected as stiff cells.

### Animal experiments protocol

All studies involving mice were approved by the Animal Care and Use Committee of the CAMS. All the animals were allocated randomly. For tumor formation, the isolated stiff or soft 4T1 or MCF‐7 cells were injected into the mammary fat pads of BALB/c or NSG mice, and B16 or MP‐1 cells were subcutaneously injected into the right flank of C57BL/6 or NSG mice. Mice were examined weekly for tumors by observation and palpation. Eight to twelve weeks later, the percentage of tumor formation was calculated. For the generation of lung metastatic models, C57BL/6 or NSG mice were injected with isolated soft or stiff B16 or MP‐1 cells by tail vein injection. Six weeks later, H&E and immunohistochemistry staining of lungs from mice were applied to evaluate metastatic ability.

### Analysis of cellular stiffness by atomic force microscopy

Cell stiffness was measured by using a BioScope Resolve (Bruker, Santa Barbara, USA) AFM. The cantilevers for AFM were with a nominal spring constant of 0.5 Nm‐1. The AFM imaging was recorded at room temperature and the scan rate was 1.00 Hz, with tip velocity of 100 μm/s. The Peak Force Setpoint for Feedback was set to 0.6 V. The peak force frequency and peak force amplitude were set to 1 kHz and 300 nm, respectively. Hundreds of cells were imaged and the cellular stiffness was measured and analyzed with Nanoscope Analysis 1.9 Software (Bruker, Karlsruhe, Germany).

### Real‐time PCR

TRIzol (Invitrogen, Cat. 15596018) was used to extract total RNA from cells which was then transcribed to cDNA using a high capacity cDNA reverse transcription kit (Applied Biosystems, Cat. 4368813). Real‐time PCR was performed using ABI stepone plus (Applied Biosystems, CA, USA). The primer sequences are shown as follows: *Gapdh*, AGGTCGGTGTGAACGGATTTG (sense) and TGTAGACCATGTAGTTG AGGTCA (antisense); *GAPDH*, ACAACTTTGGTATCGTGGAAGG (sense) and GCCATCACGCCACAGTTTC (antisense); *Bcl9l*, CGCGAGAGGAGTGTGTCTG (sense) and CCATTCGTCCCCACTGTACG (antisense); *BCL9L*, TCTCGCCTAGC AACTCAAGTC (sense) and GAGCACCATTCGTCCCCAC (antisense); *Ctnnb1*, ATGGAGCCGGACAGAAAAGC (sense) and CTTGCCACTCAGGGAAGGA (antisense); *CTNNB1*, CATCTACACAGTTTGATGCTGCT (sense) and GCAGTTTT GTCAGTTCAGGGA (antisense). Values are means ± SD from three independent experiments which were performed in duplicate. Statistical comparisons among groups were performed using one‐way ANOVA or Student’s *t*‐test. Values of all parameters were considered statistically significant difference at a value of *P* < 0.05.

### Generation of CRISPR‐Cas9 knockout cell lines

For construction of the stable knockdown of BCL9L‐B16, 4T1, or MCF‐7 cells, the following SGRNAs targeting BCL9L were used: SGGFP, CACCGGGGCGAGGAG CTGTTCACCG (sense) and AAACCGGTGAACAGCTC CTCGCCCC (antisense); *BCL9L*‐SGRNA1, TGACCAATCATGGCAAGACA (sense) and TGTCTTGCCAT GATTGGTCA (antisense); *BCL9L*‐SGRNA2, CCAAGGACCCACCTGCAACG (sense) and CGTTGCAGGTGGGTCCTTGG (antisense); *Bcl9l*‐SGRNA1, CCAGG TTACCCCACCCCAGG (sense) and CCTGGGGTGGGGTAACC TGG (antisense); *Bcl9l*‐SGRNA2, AGTCCACCGCTGTCCCCTCG (sense) and CGAGGGGACAGC GGTGGACT (antisense). These SGRNAs were cloned into the pL‐CRISPR.EFS.RFP vector plasmid (addgene, #57819) and transfected HEK 293T cells together with the packing plasmids psPAX2 and pMD2.G. Forty‐eight hours later, the lentivirus was harvested and concentrated to infect B16, 4T1, or MCF‐7 cells together with polybrene at a final concentration of 8 μg/ml. Two days later, RFP‐positive cells were sorted by flow cytometry using the BD Biosciences FACSAria III. The candidate knockout cells were verified by Western blot.

### Western blotting

Cells were collected, lysed in M2 lysis buffer, and sonicated. The protein concentrations were determined by the BCA kit (Applygen Technologies Inc., China). Then, the protein was run on an SDS–PAGE gel and transferred to nitrocellulose membrane. Nitrocellulose membranes were blocked in 5% bovine serum albumin (BSA) and probed with antibodies overnight: anti‐β‐actin (Cell signaling technology, Cat.: 3700S; clone: 8H10D10); BCL9L (Thermo fisher, Cat.: PA5‐21111), and anti‐β‐catenin (Cell signaling technology, Cat.: 8480S; clone: D10A8). Secondary antibodies conjugated to horseradish peroxidase were followed by enhanced chemiluminescence (Thermo fisher, MA). Results were confirmed by at least three independent experiments.

### Hoechst 33342 staining and ALDH^+^, CD133^+^, or SP tumor cells sorting

The tumor cells were resuspended (10^6^ cells/ml) in Hank’s balanced salt solution (HBSS) and incubated for 30 min at 37°C with 1 μg/ml Hoechst 33342 (Thermo fisher, Cat.: H1399). Then, these suspended cells were stained with PE‐conjugated anti‐CD133 antibody (1:100; Biolegend, Cat.: 393904) at room temperature for 30 min. After being washed with ice‐cold HBSS, these cells were filtered through a 40 μm cell strainer (JET Biofil, China) to obtain a single‐cell suspension for sorting on a BD FACSARIA III (BD Bioscience, NJ, USA). The ALDH^+^ cells were sorted by using ALDEFLUOR Kit (Stemcell Technologies, Cat.: 01700) according to the supplier’s instruction.

### Immunofluorescence

Cells were fixed in 4% polyoxymethylene and permeabilized with 0.5% Triton X‐100 at 4°C for 10 min. Then, these cells were blocked with 5% BSA for 20 min at room temperature. After incubation with anti‐β‐catenin (1:1,000, Cell signaling technology, Cat.: 8480S) or BCL9L (1:1,000, Thermo fisher, Cat.: PA5‐21111) at 4°C overnight, cells were washed and incubated sequentially with HRP‐conjugated secondary antibodies for 1 h at room temperature. At last, the slides were counterstained with DAPI and mounted for confocal analysis. The intensity of immunofluorescence was analyzed by ImageJ 9.0 software.

### Wound‐healing assay

The monolayer cells in 6‐well plate were scraped in a straight line with a 10‐μl pipette tip to produce a wound. After being washed with ice‐cold HBSS, cells were incubated in medium containing 1% fetal bovine serum (FBS). Photographs of the scratch were taken at 0 and 24 h after wounding using the Olympus‐inverted microscope. Gap width at 0 h was set to 1. Multiple defined sites along the scratch were measured. Data are shown as the average of three independent experiments.

### Migration and invasion assay

For cell migration assay, 2–10 × 10^4^ cells in 200 μl serum‐free medium were plated in an 8.0‐μm, 24‐well Hanging Insert (Millipore, Cat.: MCEP24H48), and 400 µl medium containing 10% FBS was added to the lower chamber of a transwell dish. After incubation for 24–48 h, non‐migrating cells were removed from the upper surface of the membrane, and cells that migrated through the 8 µm pore membrane were fixed with 4% paraformaldehyde and stained with 0.5% crystal violet. The migrating cells were photographed and counted using an inverted microscope. For the cell invasion assay, ECMatrix™‐coated chambers (Millipore, Cat.: ECM550) were used according to the supplier’s instruction. Briefly, the cell suspension containing 0.5–1.0 × 10^6^ cells/ml in serum‐free media was added to each insert, and 500 µl medium containing 10% FBS was added to the lower chamber of a transwell dish. After incubation for 24–72 h, the non‐invading cells as well as the ECMatrix gel were removed gently from the interior of the inserts by using a cotton‐tipped swab. The invading cells were fixed with 4% paraformaldehyde and stained with 0.5% crystal violet for 20 min, and photographed and counted using an inverted microscope.

### Isolation of primary tumor cells

The surgically removed tumor tissues were cut into small pieces of 1–3 mm, minced, and incubated for 1 h at 37°C under continuous rotation with RPMI 1640 medium supplemented with collagenase type IV (Sigma, 32 µg/ml, Cat. C5138), hyaluronidase (Sigma, 500 µg/ml, Cat. H1136), and DNAase I (Sigma, 5 µg/ml, Cat. 11284932001). After the digestion, single‐cell suspensions were obtained by filtering through a 70‐µm cell strainer before being pelleted by centrifugation and then erythrocytes (RBC) were removed by exposed to RBC lysis buffer. Then, single‐cell suspensions were magnetically labeled using the Non‐Tumor Cell Depletion Cocktails microbeads contained in the Tumor Cell Isolation Kit (Miltenyi Biotec, Cat. 130‐108‐339), according to the supplier’s instruction. Finally, the unlabeled tumor cells flow‐through the LC column in the magnetic field of MACS^®^ Separator were collected for subsequent analysis. Tumor cells were isolated to a purity of > 95%, regardless of the starting frequency of tumor cells.

### Quantification and statistical analysis

All experiments were performed at least three times. Results are expressed as mean ± SD as indicated and analyzed by Student's *t*‐test followed by two‐tailed paired *t*‐test or Mann–Whitney test or one‐way ANOVA followed by Bonferroni or Kruskal–Wallis test as indicated. The *P* value < 0.05 was considered statistically significant. The analysis was conducted using the GraphPad 6.0 software. The survival rates were evaluated by the Log‐rank test.

## Author contributions

Project conception: BH and YYL; Experiments: JLv, YPL, FC, JLi, YZ, TZ, NZ, CL, ZW, LM, QZ, XL, ML, KT, JM, HZ, YYL, and JX; Methodology development: YPL, JLv, YYL, YF, HZ, and NW; Data analysis: BH, YPL, JLv, YYL, and JLi; Manuscript writing: BH, YYL, and JLv.

## Conflict of interest

The authors declare that they have no conflict of interest.

## Supporting information



Expanded View Figures PDF

Table EV1

Table EV2

Table EV3

Table EV4

Source Data for Expanded View

Review Process File

Source Data for Figure 5

## Data Availability

The datasets produced in this study are available in the following databases: RNA‐Seq and ATAC‐seq data: The National Genomics Data Center (NGDC) Genome Sequence Archive PRJCA003394 (https://bigd.big.ac.cn/gsa/s/3L1LdBT6).

## References

[embj2020106123-bib-0001] Al‐Hajj M , Wicha MS , Benito‐Hernandez A , Morrison SJ , Clarke MF (2003) Prospective identification of tumorigenic breast cancer cells. Proc Natl Acad Sci USA 100: 3983–3988 12629218 10.1073/pnas.0530291100 PMC153034

[embj2020106123-bib-0002] Beier D , Hau P , Proescholdt M , Lohmeier A , Wischhusen J , Oefner PJ , Aigner L , Brawanski A , Bogdahn U , Beier CP (2007) CD133+ and CD133‐ glioblastoma‐derived cancer stem cells show differential growth characteristics and molecular profiles. Cancer Res 67: 4010–4015 17483311 10.1158/0008-5472.CAN-06-4180

[embj2020106123-bib-0003] Chowdhury F , Na S , Li D , Poh Y‐C , Tanaka TS , Wang F , Wang N (2010) Material properties of the cell dictate stress‐induced spreading and differentiation in embryonic stem cells. Nat Mater 9: 82–88 19838182 10.1038/nmat2563 PMC2833279

[embj2020106123-bib-0004] Clevers H (2011) The cancer stem cell: premises, promises and challenges. Nat Med 17: 313–319 21386835 10.1038/nm.2304

[embj2020106123-bib-0005] Cross SE , Jin YS , Rao J , Gimzewski JK (2007) Nanomechanical analysis of cells from cancer patients. Nat Nanotechnol 2: 780–783 18654431 10.1038/nnano.2007.388

[embj2020106123-bib-0006] Dieter Sebastian M , Ball Claudia R , Hoffmann Christopher M , Nowrouzi A , Herbst F , Zavidij O , Abel U , Arens A , Weichert W , Brand K *et al* (2011) Distinct types of tumor‐initiating cells form human colon cancer tumors and metastases. Cell Stem Cell 9: 357–365 21982235 10.1016/j.stem.2011.08.010

[embj2020106123-bib-0007] Discher DE , Janmey P , Wang Y‐l (2005) Tissue cells feel and respond to the stiffness of their substrate. Science 310: 1139–1143 16293750 10.1126/science.1116995

[embj2020106123-bib-0008] Discher DE , Mooney DJ , Zandstra PW (2009) Growth factors, matrices, and forces combine and control stem cells. Science 324: 1673–1677 19556500 10.1126/science.1171643 PMC2847855

[embj2020106123-bib-0009] Douville J , Beaulieu R , Balicki D (2009) ALDH1 as a functional marker of cancer stem and progenitor cells. Stem Cells Dev 18: 17–25 18573038 10.1089/scd.2008.0055

[embj2020106123-bib-0010] Elosegui‐Artola A , Bazellières E , Allen MD , Andreu I , Oria R , Sunyer R , Gomm JJ , Marshall JF , Jones JL , Trepat X *et al* (2014) Rigidity sensing and adaptation through regulation of integrin types. Nat Mater 13: 631–637 24793358 10.1038/nmat3960 PMC4031069

[embj2020106123-bib-0011] Engler AJ , Sen S , Sweeney HL , Discher DE (2006) Matrix elasticity directs stem cell lineage specification. Cell 126: 677–689 16923388 10.1016/j.cell.2006.06.044

[embj2020106123-bib-0012] Erler JT , Bennewith KL , Nicolau M , Dornhöfer N , Kong C , Le QT , Chi JT , Jeffrey SS , Giaccia AJ (2006) Lysyl oxidase is essential for hypoxia‐induced metastasis. Nature 440: 1222–1226 16642001 10.1038/nature04695

[embj2020106123-bib-0013] Gilkes DM , Semenza GL , Wirtz D (2014) Hypoxia and the extracellular matrix: drivers of tumour metastasis. Nat Rev Cancer 14: 430–439 24827502 10.1038/nrc3726 PMC4283800

[embj2020106123-bib-0014] Ginestier C , Hur MH , Charafe‐Jauffret E , Monville F , Dutcher J , Brown M , Jacquemier J , Viens P , Kleer CG , Liu S *et al* (2007) ALDH1 is a marker of normal and malignant human mammary stem cells and a predictor of poor clinical outcome. Cell Stem Cell 1: 555–567 18371393 10.1016/j.stem.2007.08.014 PMC2423808

[embj2020106123-bib-0015] Golebiewska A , Brons NHC , Bjerkvig R , Niclou SP (2011) Critical appraisal of the side population assay in stem cell and cancer stem cell research. Cell Stem Cell 8: 136–147 21295271 10.1016/j.stem.2011.01.007

[embj2020106123-bib-0016] Gregorieff A , Liu Y , Inanlou MR , Khomchuk Y , Wrana JL (2015) Yap‐dependent reprogramming of Lgr5+ stem cells drives intestinal regeneration and cancer. Nature 526: 715–718 26503053 10.1038/nature15382

[embj2020106123-bib-0017] Guck J , Schinkinger S , Lincoln B , Wottawah F , Ebert S , Romeyke M , Lenz D , Erickson HM , Ananthakrishnan R , Mitchell D *et al* (2005) Optical deformability as an inherent cell marker for testing malignant transformation and metastatic competence. Biophys J 88: 3689–3698 15722433 10.1529/biophysj.104.045476 PMC1305515

[embj2020106123-bib-0018] Guo L , Ye C , Hao X , Zheng R , Ju R , Wu D , Luo L , Wang C , Li J , Yu X *et al* (2012) Carboxyamidotriazole ameliorates experimental colitis by inhibition of cytokine production, nuclear factor‐kappaB activation, and colonic fibrosis. J Pharmacol Exp Ther 342: 356–365 22553216 10.1124/jpet.112.192849

[embj2020106123-bib-0019] Hope KJ , Jin L , Dick JE (2004) Acute myeloid leukemia originates from a hierarchy of leukemic stem cell classes that differ in self‐renewal capacity. Nat Immunol 5: 738–743 15170211 10.1038/ni1080

[embj2020106123-bib-0020] Hosokawa M , Hayata T , Fukuda Y , Arakaki A , Yoshino T , Tanaka T , Matsunaga T (2010) Size‐selective microcavity array for rapid and efficient detection of circulating tumor cells. Anal Chem 82: 6629–6635 20583799 10.1021/ac101222x

[embj2020106123-bib-0021] Hvichia GE , Parveen Z , Wagner C , Janning M , Quidde J , Stein A , Müller V , Loges S , Neves RP , Stoecklein NH *et al* (2016) A novel microfluidic platform for size and deformability based separation and the subsequent molecular characterization of viable circulating tumor cells. Int J Cancer 138: 2894–2904 26789903 10.1002/ijc.30007 PMC5069649

[embj2020106123-bib-0022] Iinuma H , Watanabe T , Mimori K , Adachi M , Hayashi N , Tamura J , Matsuda K , Fukushima R , Okinaga K , Sasako M *et al* (2011) Clinical significance of circulating tumor cells, including cancer stem‐like cells, in peripheral blood for recurrence and prognosis in patients with Dukes' stage B and C colorectal cancer. J Clin Oncol 29: 1547–1555 21422427 10.1200/JCO.2010.30.5151

[embj2020106123-bib-0023] Irianto J , Pfeifer CR , Xia Y , Discher DE (2016) SnapShot: mechanosensing matrix. Cell 165: 1820–1820.e1821 27315485 10.1016/j.cell.2016.06.002 PMC5341690

[embj2020106123-bib-0024] Kaiser J (2015) The cancer stem cell gamble. Science 347: 226–229 25593170 10.1126/science.347.6219.226

[embj2020106123-bib-0025] Kapucuoğlu N , Bozkurt KK , Başpınar Ş , Koçer M , Eroğlu HE , Akdeniz R , Akçil M (2015) The clinicopathological and prognostic significance of CD24, CD44, CD133, ALDH1 expressions in invasive ductal carcinoma of the breast: CD44/CD24 expression in breast cancer. Pathol Res Pract 211: 740–747 26298632 10.1016/j.prp.2015.05.011

[embj2020106123-bib-0026] Keysar SB , Jimeno A (2010) More than markers: biological significance of cancer stem cell‐defining molecules. Mol Cancer Ther 9: 2450–2457 20716638 10.1158/1535-7163.MCT-10-0530 PMC3618879

[embj2020106123-bib-0027] Lapidot T , Sirard C , Vormoor J , Murdoch B , Hoang T , Caceres‐Cortes J , Minden M , Paterson B , Caligiuri MA , Dick JE (1994) A cell initiating human acute myeloid leukaemia after transplantation into SCID mice. Nature 367: 645–648 7509044 10.1038/367645a0

[embj2020106123-bib-0028] Liu J , Tan Y , Zhang H , Zhang Y , Xu P , Chen J , Poh Y‐C , Tang K , Wang N , Huang B (2012) Soft fibrin gels promote selection and growth of tumorigenic cells. Nat Mater 11: 734–741 22751180 10.1038/nmat3361 PMC3405191

[embj2020106123-bib-0029] Liu Y , Liang X , Dong W , Fang Y , Lv J , Zhang T , Fiskesund R , Xie J , Liu J , Yin X *et al* (2018) Tumor‐repopulating cells induce PD‐1 expression in CD8(+) T cells by transferring kynurenine and AhR activation. Cancer Cell 33: 480–494.e487 29533786 10.1016/j.ccell.2018.02.005

[embj2020106123-bib-0030] Lugli A , Iezzi G , Hostettler I , Muraro MG , Mele V , Tornillo L , Carafa V , Spagnoli G , Terracciano L , Zlobec I (2010) Prognostic impact of the expression of putative cancer stem cell markers CD133, CD166, CD44s, EpCAM, and ALDH1 in colorectal cancer. Br J Cancer 103: 382–390 20606680 10.1038/sj.bjc.6605762 PMC2920016

[embj2020106123-bib-0031] Medema JP (2013) Cancer stem cells: the challenges ahead. Nat Cell Biol 15: 338–344 23548926 10.1038/ncb2717

[embj2020106123-bib-0032] Miller TJ , McCoy MJ , Hemmings C , Bulsara MK , Iacopetta B , Platell CF (2017) The prognostic value of cancer stem‐like cell markers SOX2 and CD133 in stage III colon cancer is modified by expression of the immune‐related markers FoxP3, PD‐L1 and CD3. Pathology 49: 721–730 29102042 10.1016/j.pathol.2017.08.007

[embj2020106123-bib-0033] Mohamed H , Murray M , Turner JN , Caggana M (2009) Isolation of tumor cells using size and deformation. J Chromatogr A 1216: 8289–8295 19497576 10.1016/j.chroma.2009.05.036

[embj2020106123-bib-0034] O'Brien CA , Pollett A , Gallinger S , Dick JE (2007) A human colon cancer cell capable of initiating tumour growth in immunodeficient mice. Nature 445: 106–110 17122772 10.1038/nature05372

[embj2020106123-bib-0035] Ondeck MG , Kumar A , Placone JK , Plunkett CM , Matte BF , Wong KC , Fattet L , Yang J , Engler AJ (2019) Dynamically stiffened matrix promotes malignant transformation of mammary epithelial cells via collective mechanical signaling. Proc Natl Acad Sci USA 116: 3502–3507 30755531 10.1073/pnas.1814204116 PMC6397509

[embj2020106123-bib-0036] Paszek MJ , Zahir N , Johnson KR , Lakins JN , Rozenberg GI , Gefen A , Reinhart‐King CA , Margulies SS , Dembo M , Boettiger D *et al* (2005) Tensional homeostasis and the malignant phenotype. Cancer Cell 8: 241–254 16169468 10.1016/j.ccr.2005.08.010

[embj2020106123-bib-0037] Plodinec M , Loparic M , Monnier CA , Obermann EC , Zanetti‐Dallenbach R , Oertle P , Hyotyla JT , Aebi U , Bentires‐Alj M , Lim RY *et al* (2012) The nanomechanical signature of breast cancer. Nat Nanotechnol 7: 757–765 23085644 10.1038/nnano.2012.167

[embj2020106123-bib-0038] Quintana E , Shackleton M , Sabel MS , Fullen DR , Johnson TM , Morrison SJ (2008) Efficient tumour formation by single human melanoma cells. Nature 456: 593–598 19052619 10.1038/nature07567 PMC2597380

[embj2020106123-bib-0039] Rankin EB , Giaccia AJ (2016) Hypoxic control of metastasis. Science 352: 175–180 27124451 10.1126/science.aaf4405 PMC4898055

[embj2020106123-bib-0040] Schatton T , Murphy GF , Frank NY , Yamaura K , Waaga‐Gasser AM , Gasser M , Zhan Q , Jordan S , Duncan LM , Weishaupt C *et al* (2008) Identification of cells initiating human melanomas. Nature 451: 345–349 18202660 10.1038/nature06489 PMC3660705

[embj2020106123-bib-0041] Shmelkov SV , Butler JM , Hooper AT , Hormigo A , Kushner J , Milde T , St Clair R , Baljevic M , White I , Jin DK *et al* (2008) CD133 expression is not restricted to stem cells, and both CD133+ and CD133‐ metastatic colon cancer cells initiate tumors. J Clin Invest 118: 2111–2120 18497886 10.1172/JCI34401 PMC2391278

[embj2020106123-bib-0042] Singh SK , Hawkins C , Clarke ID , Squire JA , Bayani J , Hide T , Henkelman RM , Cusimano MD , Dirks PB (2004) Identification of human brain tumour initiating cells. Nature 432: 396–401 15549107 10.1038/nature03128

[embj2020106123-bib-0043] Stavropoulou V , Kaspar S , Brault L , Sanders MA , Juge S , Morettini S , Tzankov A , Iacovino M , Lau IJ , Milne TA *et al* (2016) MLL‐AF9 expression in hematopoietic stem cells drives a highly invasive AML expressing EMT‐related genes linked to poor outcome. Cancer Cell 30: 43–58 27344946 10.1016/j.ccell.2016.05.011

[embj2020106123-bib-0044] Swaminathan V , Mythreye K , O'Brien ET , Berchuck A , Blobe GC , Superfine R (2011) Mechanical stiffness grades metastatic potential in patient tumor cells and in cancer cell lines. Cancer Res 71: 5075–5080 21642375 10.1158/0008-5472.CAN-11-0247 PMC3220953

[embj2020106123-bib-0045] Tan Y , Tajik A , Chen J , Jia Q , Chowdhury F , Wang L , Chen J , Zhang S , Hong Y , Yi H *et al* (2014) Matrix softness regulates plasticity of tumour‐repopulating cells via H3K9 demethylation and Sox2 expression. Nat Commun 5: 4619 25099074 10.1038/ncomms5619 PMC4133791

[embj2020106123-bib-0046] Tang K , Yu Y , Zhu L , Xu P , Chen J , Ma J , Zhang H , Fang H , Sun W , Zhou L *et al* (2019) Hypoxia‐reprogrammed tricarboxylic acid cycle promotes the growth of human breast tumorigenic cells. Oncogene 38: 6970–6984 31409901 10.1038/s41388-019-0932-1

[embj2020106123-bib-0047] Taussig DC , Miraki‐Moud F , Anjos‐Afonso F , Pearce DJ , Allen K , Ridler C , Lillington D , Oakervee H , Cavenagh J , Agrawal SG *et al* (2008) Anti‐CD38 antibody‐mediated clearance of human repopulating cells masks the heterogeneity of leukemia‐initiating cells. Blood 112: 568–575 18523148 10.1182/blood-2007-10-118331

[embj2020106123-bib-0048] Taussig DC , Vargaftig J , Miraki‐Moud F , Griessinger E , Sharrock K , Luke T , Lillington D , Oakervee H , Cavenagh J , Agrawal SG *et al* (2010) Leukemia‐initiating cells from some acute myeloid leukemia patients with mutated nucleophosmin reside in the CD34(‐) fraction. Blood 115: 1976–1984 20053758 10.1182/blood-2009-02-206565 PMC2837317

[embj2020106123-bib-0049] Urbanska M , Winzi M , Neumann K , Abuhattum S , Rosendahl P , Müller P , Taubenberger A , Anastassiadis K , Guck J (2017) Single‐cell mechanical phenotype is an intrinsic marker of reprogramming and differentiation along the mouse neural lineage. Development 144: 4313–4321 29183942 10.1242/dev.155218

[embj2020106123-bib-0050] van Tienen LM , Mieszczanek J , Fiedler M , Rutherford TJ , Bienz M (2017) Constitutive scaffolding of multiple Wnt enhanceosome components by Legless/BCL9. Elife 6: e20882 28296634 10.7554/eLife.20882 PMC5352222

[embj2020106123-bib-0051] Wakamatsu Y , Sakamoto N , Oo HZ , Naito Y , Uraoka N , Anami K , Sentani K , Oue N , Yasui W (2012) Expression of cancer stem cell markers ALDH1, CD44 and CD133 in primary tumor and lymph node metastasis of gastric cancer. Pathol Int 62: 112–119 22243781 10.1111/j.1440-1827.2011.02760.x

[embj2020106123-bib-0052] Wang N , Butler J , Ingber D (1993) Mechanotransduction across the cell surface and through the cytoskeleton. Science 260: 1124–1127 7684161 10.1126/science.7684161

[embj2020106123-bib-0053] Wang T , Shigdar S , Gantier MP , Hou Y , Wang L , Li Y , Shamaileh HA , Yin W , Zhou S‐F , Zhao X *et al* (2015) Cancer stem cell targeted therapy: progress amid controversies. Oncotarget 6: 44191–44206 26496035 10.18632/oncotarget.6176 PMC4792551

[embj2020106123-bib-0054] Wei SC , Fattet L , Tsai JH , Guo Y , Pai VH , Majeski HE , Chen AC , Sah RL , Taylor SS , Engler AJ *et al* (2015) Matrix stiffness drives epithelial‐mesenchymal transition and tumour metastasis through a TWIST1‐G3BP2 mechanotransduction pathway. Nat Cell Biol 17: 678–688 25893917 10.1038/ncb3157 PMC4452027

[embj2020106123-bib-0055] Wu P‐H , Aroush DR‐B , Asnacios A , Chen W‐C , Dokukin ME , Doss BL , Durand‐Smet P , Ekpenyong A , Guck J , Guz NV *et al* (2018) A comparison of methods to assess cell mechanical properties. Nat Methods 15: 491–498 29915189 10.1038/s41592-018-0015-1 PMC6582221

[embj2020106123-bib-0056] Xu W , Mezencev R , Kim B , Wang L , McDonald J , Sulchek T (2012) Cell stiffness is a biomarker of the metastatic potential of ovarian cancer cells. PLoS One 7: e46609 23056368 10.1371/journal.pone.0046609 PMC3464294

[embj2020106123-bib-0057] Zhang W , Kai K , Choi DS , Iwamoto T , Nguyen YH , Wong H , Landis MD , Ueno NT , Chang J , Qin L (2012) Microfluidics separation reveals the stem‐cell‐like deformability of tumor‐initiating cells. Proc Natl Acad Sci USA 109: 18707–18712 23112172 10.1073/pnas.1209893109 PMC3503214

