# Supplementary material for: Cell softness regulates tumorigenicity and stemness of cancer cells
Source: EMBO J. 2020 Dec 4;40(2):e106123. doi: 10.15252/embj.2020106123 (PMC7809788; doi:10.15252/embj.2020106123)
Supplement: Supplementary file 1 — Expanded View Figures PDF [file EMBJ-40-e106123-s001.pdf]

Expanded View Figures

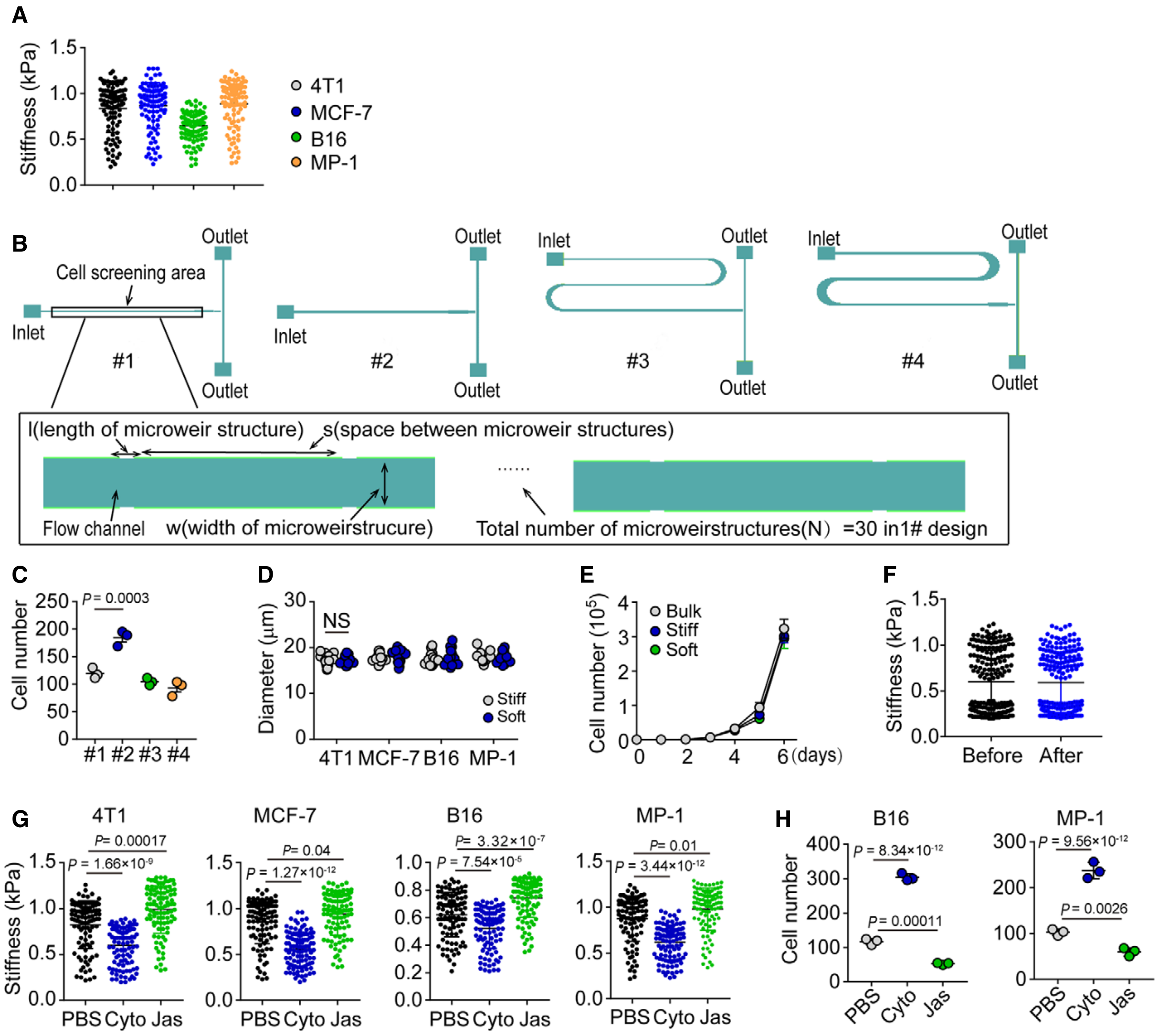

Figure EV1. Soft tumor cells are isolated by microfluidic chip.

- A The cellular stiffness of 4T1, MCF-7, B16F1, or MP-1 was detected by AFM. *n* = 100.
- B The schematic illustration of PDMS microfluidic chip fabrication for four different types. The lower is the magnification of the indicated microfluidic channel.
- C The number of soft B16 cells isolated from four different microfluidic chips at the same flow flux of 13 μl/min for 10 min (cell density, 1 × 10^4 cells/ml).
- D The size of separated stiff and soft cells from 4T1, MCF-7, B16, or MP-1 bulk cells was measured. *n* = 10.
- E The bulk, stiff, or soft B16 cells were cultured in 6-well plate for the indicated time periods.
- F The soft and stiff 4T1 cells were isolated by microfluidic chip (before) and then mixed together to be separated again by microfluidic chip (after). The stiffness of 4T1 cells from before and after re-separation was detected by AFM. *n* = 100.
- G The stiffness of 4T1, MCF-7, B16, and MP-1 cells treated with Cyto (5 μM) or Jas (50 nM) for 4 h or 12 h was measured by AFM. *n* = 100.
- H The soft tumor cells were isolated by microfluidic chip from B16 or MP-1 cells pre-treated with Cyto (5 μM) or Jas (50 nM) for 4 h or 12 h. The number of soft tumor cells was counted. *n* = 3.

Data information: N.S., no significant difference. Kruskal–Wallis test (G), Bonferroni test (C, D and H). The data represent mean ± SD of three independent experiments.

© 2020 The Authors

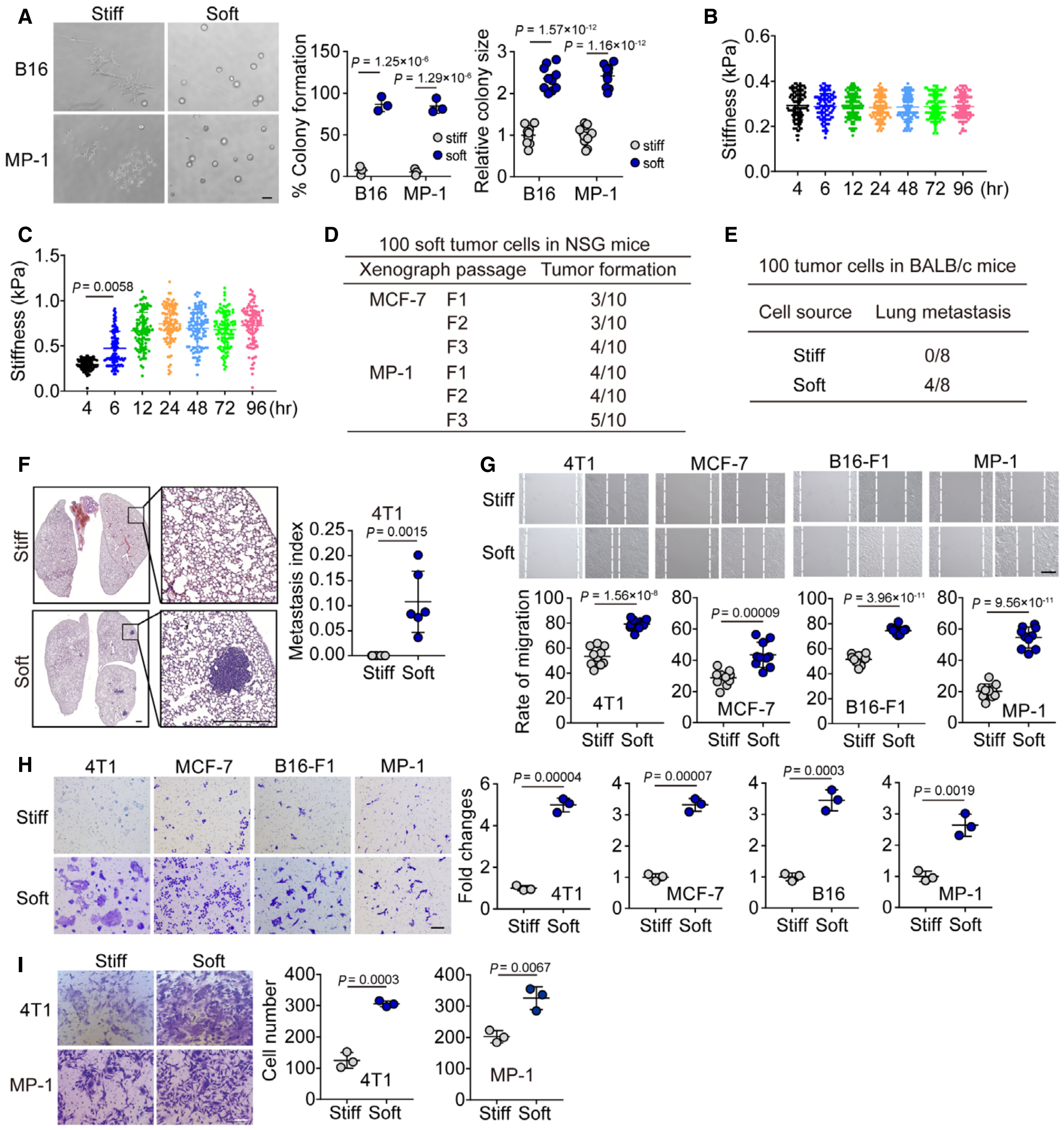

Figure EV2.

 © 2020 The Authors

Figure EV2. Soft tumor cells have a greater ability to migrate and invade compared with the stiff cells.

A The stiff or soft B16 or MP-1 cells were isolated by microfluidic chip, and then 500 soft or stiff cells were seeded in a 90 Pa soft 3D fibrin gel for 3 days. The percentage of colony formation was calculated, and the colony size was recorded. Scale bar, 100 μm. $n = 3$ for colony number and 10 for colony size.

B, C Soft MCF-7 cells isolated by the microfluidic chip were cultured in soft 3D fibrin gel (B) or rigid flask (C) for 4, 6, 12, 24, 48, 72, and 96 h. Stiffness of the cells was determined by AFM. $n = 100$.

D The tumor-forming capacity from primary xenografts (F1) and tumors passaged into secondary (F2) and tertiary (F3) recipients induced by injecting 100 soft MCF-7 or MP-1 cells into NSG mice. $n = 10$.

E, F 100 stiff or soft 4T1 cells were injected into the mammary fat pads of BALB/c (E) or NSG (F) mice ($n = 8$). Eight weeks later, the lung metastasis was counted (E) and analyzed by H&E staining (F), $n = 6$ mice with metastatic tumor. Scale bar, 0.5 mm.

G The soft or stiff B16-F1, MP-1, 4T1, or MCF-7 cells were grown to confluence. Then, cells were scratched and wound closure was recorded at 24 h by phase contrast microscopy. Representative images of are shown. Wound closure was calculated using ImageJ software and expressed as a percentage of the initial scratched area. Scale bar, 250 μm. $n = 10$.

H The stiff or soft 4T1, MCF-7, B16-F1, or MP-1 cells were added into the hanging insert for 24 h (4T1) or 48 h (MCF-7, B16-F1, or MP-1). Then, the non-migrating cells were removed from the upper surface of the membrane, and cells that migrated through the 8 μm pore membrane were fixed and stained with 0.1% crystal violet. Scale bar, 50 μm. $n = 3$.

I The stiff or soft 4T1 or MP-1 cells were added to the top of a matrigel invasion chamber for 24 h or 48 h. Then, the non-invasive cells were removed from the upper surface of the membrane, and the invading cells were fixed and stained with 0.1% crystal violet. Representative pictures of invading cells and the quantification of cell invasion are shown. Scale bar, 50 μm. $n = 3$.

Data information: Two-tailed paired Student's *t*-test (A and F–I), Kruskal–Wallis test (C). The data represent mean ± SD.

Source data are available online for this figure.

© 2020 The Authors

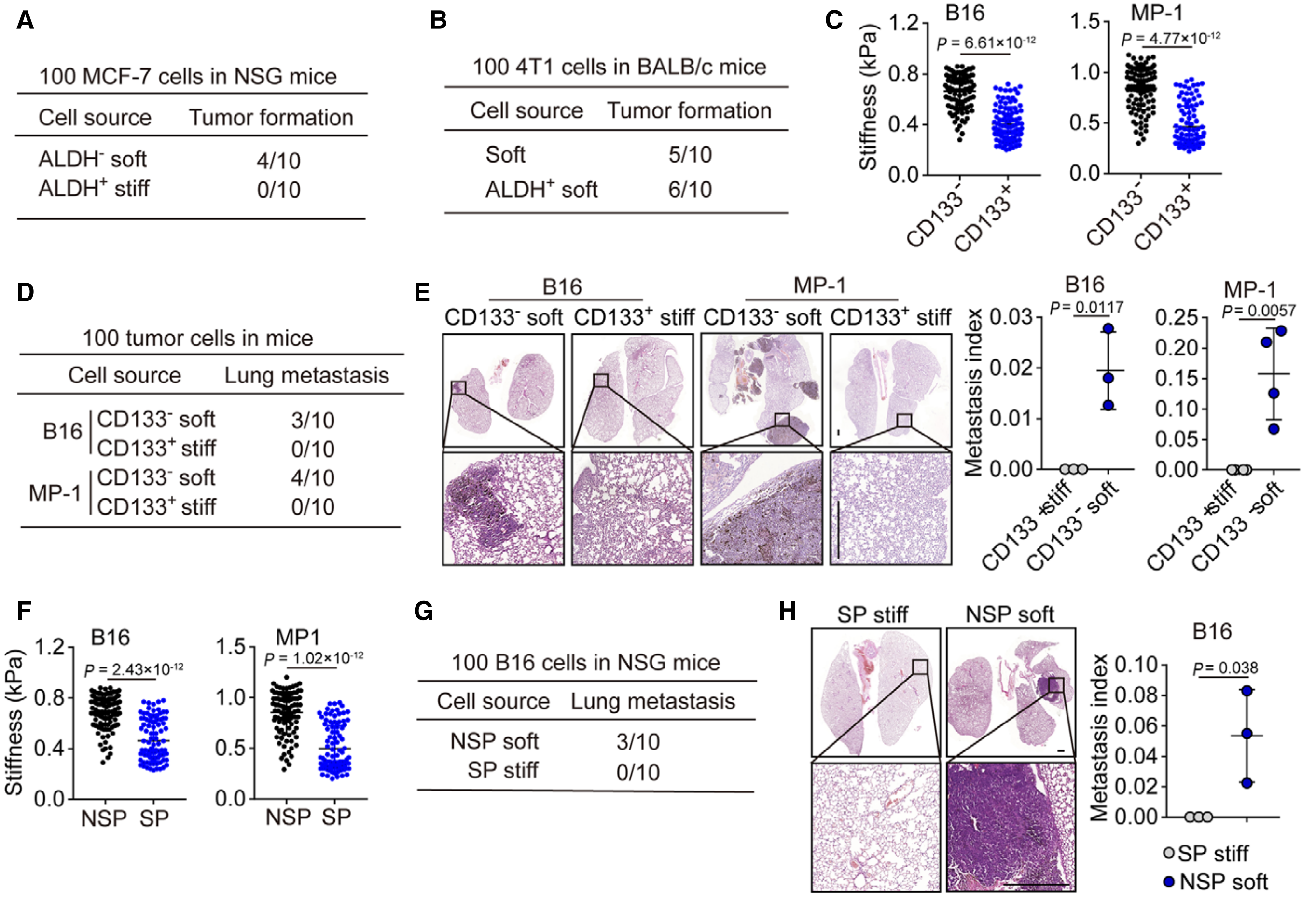

Figure EV3. Mechanical softness is the key characteristic of tumorigenic cells.

A The ALDH⁻, or ALDH⁺ stiff, or soft MCF-7 cells were injected into the mammary fat pads of NSG mice (100 cells/mouse). The tumor formation was recorded. n = 10.

B The same as (A), except that soft 4T1 or ALDH⁺ soft 4T1 cells were injected into BALB/c mice. n = 10.

C The stiffness of CD133⁻ or CD133⁺ B16 or MP-1 cells was measured by AFM. n = 100.

D, E The 100 stiff CD133⁺ or soft CD133⁻ B16 or MP-1 cells were injected into the NSG mice by tail vein injection. Six weeks later, mice were sacrificed and the lung sections were H&E stained. The mice with lung metastasis were recorded (D). The metastatic micronodules in the lung were counted, and the metastasis index was calculated (E). Scale bar, 0.5 mm. n = 3 (B16) or 4 (MP-1) mice with metastatic tumor.

F The stiffness of the side population (SP) or non-SP (NSP) B16 or MP-1 cells were detected by AFM. n = 100.

G, H The 100 soft NSP or stiff SP-B16 cells were injected i.v. into NSG mice for 6 weeks. The tumor formation in the lungs was recorded (G). The lung sections perform H&E stained. The metastatic micronodules in the lung were counted, and the metastasis index was calculated (H). Scale bar, 0.5 mm. n = 3 mice with metastatic tumor.

Data information: Mann–Whitney test (C and F), paired Student's t-test (E and H). The data represent mean ± SD.

© 2020 The Authors

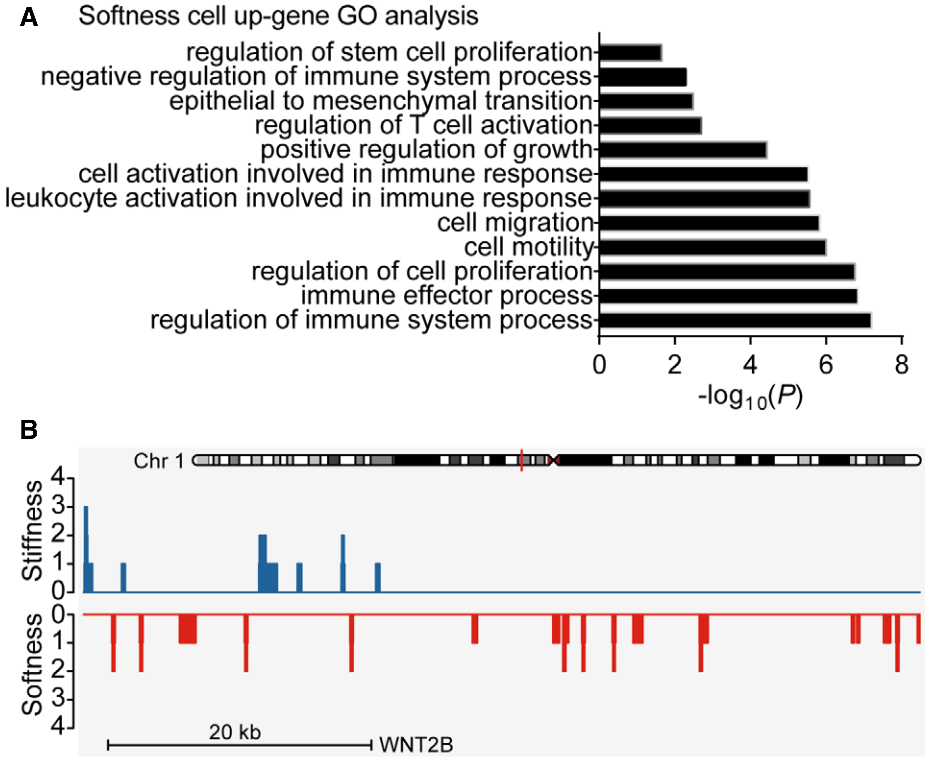

Figure EV4. RNA-seq and ATAC analysis of stiff, soft, and ALDH+ cells (CSC).

A The GO analysis of softness cells' upregulated genes was performed.
B ATAC-seq tracks of WNT2B in stiff or soft cells.

Figure EV5. BCL9L participates in regulating the stemness of soft tumor cells.

A The mRNA expression of BCL9L in stiff or soft B16, MP-1, 4T1, or MCF-7 cells was determined by real-time PCR. $n = 3$.
B, C The stiff or soft B16, MP-1, 4T1, or MCF-7 cells were stained with anti-BCL9L (B) or anti-β-catenin (C) and DAPI and observed under confocal microscope. The relative intensity was quantified. Scale bar, 10 μm. $n = 10$.
D The expression of nuclear β-catenin in stiff or soft 4T1, MCF-7, B16, or MP-1 cells was determined by Western blot.
E SGGFP, *BCL9L*-SGs- 4T1, MCF-7, B16, or MP-1 cells were stained with anti-β-catenin antibody and observed under confocal microscope. The relative intensity was quantified. Scale bar, 10 μm. $n = 10$.
F 500 soft SGGFP or *BCL9L*-SGs-4T1, MCF-7, B16, or MP-1 cells were seeded in 90 Pa soft 3D fibrin gel for the indicated time periods. The colony size was measured. The colony size in D0 (day 0) was set to 1. $n = 3$.
G The mRNA expression of Nestin, Nanog, OCT3/4, CD133, or SOX2 in stiff or soft SGGFP or *BCL9L*-SGs- B16, MP-1, 4T1, or MCF-7 cells was detected by real-time PCR. $n = 3$.
H The 100 soft SGGFP or *BCL9L*-SGs-4T1 cells were injected into the mammary fat pads of NSG mice for 8 weeks. Then, mice were sacrificed and the lung sections were performed H&E stained. The metastasis index was calculated. $n = 5$ mice with metastatic tumor. Scale bar, 0.5 mm.
I The Transwell invasion assay from soft SGGFP- or *BCL9L*-SGs- 4T1 or MP-1 cells. The number of invading cells was calculated from 3 fields. $n = 3$. Scale bar, 50 μm.
J Overall survival compared with the BCL9L level in patients with liver (LIHC, $n = 182$), lung (LUAD, $n = 240$), or pancreatic (PAAD, $n = 90$) cancer.

Data information: Paired Student's *t*-test (A–C), Bonferroni test (E–I) or Log-rank survival analysis (J). The data represent mean ± SD.
Source data are available online for this figure.

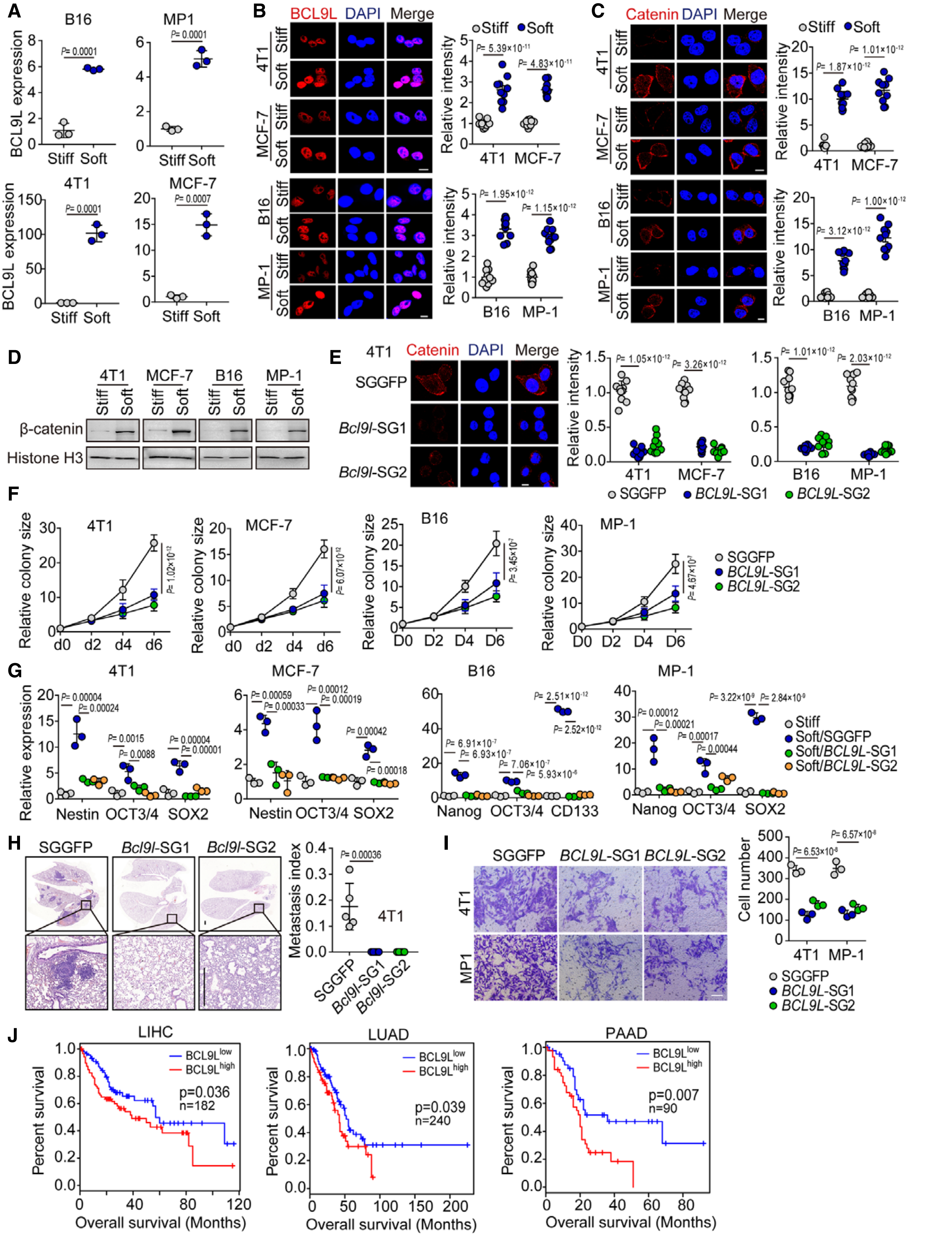

Figure EV5.

 © 2020 The Authors
