# Supplementary material for: Cell softness regulates tumorigenicity and stemness of cancer cells
Source: EMBO J. 2020 Dec 4;40(2):e106123. doi: 10.15252/embj.2020106123 (PMC7809788; doi:10.15252/embj.2020106123)
Supplement: Supplementary file 2 — Table EV1 [file EMBJ-40-e106123-s002.docx]

**Table. EV1. The characteristics of four kinds of microfluidic chip**

|  | Microfluidic chip | | | |
| --- | --- | --- | --- | --- |
|  | 1# | 2# | 3# | 4# |
| Width of inlet (μm) | 80 | 160 | 80 | 160 |
| Width of outlet (μm) | 60 | 100 | 60 | 100 |
| Gap between two ridges (μm) | 280 | 260 | 280 | 260 |
| channel length (mm) | 11.44 | 11.44 | 33.16 | 33.16 |
