# Supplementary material for: Cell softness regulates tumorigenicity and stemness of cancer cells
Source: EMBO J. 2020 Dec 4;40(2):e106123. doi: 10.15252/embj.2020106123 (PMC7809788; doi:10.15252/embj.2020106123)
Supplement: Supplementary file 3 — Table EV2 [file EMBJ-40-e106123-s003.docx]

**Table EV2. Clinical information of melanoma patients**

| Patients NO. | Gender | Age | Status | Sample |
| --- | --- | --- | --- | --- |
| 1 | Male | 54 | Newly diagnosed | Tumor tissue |
| 2 | Male | 64 | Newly diagnosed | Tumor tissue |
| 3 | Female | 55 | Newly diagnosed | Tumor tissue |
| 4 | Female | 44 | Newly diagnosed | Tumor tissue |
| 5 | Female | 64 | Newly diagnosed | Tumor tissue |
| 6 | Female | 53 | Newly diagnosed | Tumor tissue |
| 7 | Male | 42 | Newly diagnosed | Tumor tissue |
| 8 | Male | 35 | Newly diagnosed | Tumor tissue |
| 9 | Male | 77 | Newly diagnosed | Tumor tissue |
| 10 | Male | 44 | Newly diagnosed | Tumor tissue |
| 11 | Male | 64 | Newly diagnosed | Tumor tissue |
| 12 | Female | 66 | Newly diagnosed | Tumor tissue |
| 13 | Female | 69 | Newly diagnosed | Tumor tissue |
| 14 | Female | 47 | Newly diagnosed | Tumor tissue |
| 15 | Male | 63 | Newly diagnosed | Tumor tissue |
| 16 | Male | 56 | Newly diagnosed | Tumor tissue |
| 17 | Male | 58 | Newly diagnosed | Tumor tissue |
| 18 | Male | 55 | Newly diagnosed | Tumor tissue |
