# Supplementary material for: Cell softness regulates tumorigenicity and stemness of cancer cells
Source: EMBO J. 2020 Dec 4;40(2):e106123. doi: 10.15252/embj.2020106123 (PMC7809788; doi:10.15252/embj.2020106123)
Supplement: Supplementary file 4 — Table EV3 [file EMBJ-40-e106123-s004.docx]

**Table EV3. Clinical information of breast cancer patients**

| Patients NO. | Gender | Age | Status | Sample |
| --- | --- | --- | --- | --- |
| 1 | Female | 58 | Newly diagnosed | Tumor tissue |
| 2 | Female | 57 | Newly diagnosed | Tumor tissue |
| 3 | Female | 29 | Newly diagnosed | Tumor tissue |
| 4 | Female | 44 | Newly diagnosed | Tumor tissue |
| 5 | Female | 55 | Newly diagnosed | Tumor tissue |
| 6 | Female | 51 | Newly diagnosed | Tumor tissue |
| 7 | Female | 50 | Newly diagnosed | Tumor tissue |
| 8 | Female | 69 | Newly diagnosed | Tumor tissue |
| 9 | Female | 46 | Newly diagnosed | Tumor tissue |
| 10 | Female | 43 | Newly diagnosed | Tumor tissue |
| 11 | Female | 61 | Newly diagnosed | Tumor tissue |
