# Supplementary material for: Cell softness regulates tumorigenicity and stemness of cancer cells
Source: EMBO J. 2020 Dec 4;40(2):e106123. doi: 10.15252/embj.2020106123 (PMC7809788; doi:10.15252/embj.2020106123)
Supplement: Supplementary file 5 — Table EV4 [file EMBJ-40-e106123-s005.docx]

**Table EV4. Clinical information of colon cancer patients**

| Patients NO. | Gender | Age | Status | Sample |
| --- | --- | --- | --- | --- |
| 1 | Female | 64 | Newly diagnosed | Tumor tissue |
| 2 | Male | 56 | Newly diagnosed | Tumor tissue |
| 3 | Female | 54 | Newly diagnosed | Tumor tissue |
| 4 | Male | 80 | Newly diagnosed | Tumor tissue |
| 5 | Male | 74 | Newly diagnosed | Tumor tissue |
| 6 | Male | 72 | Newly diagnosed | Tumor tissue |
| 7 | Female | 53 | Newly diagnosed | Tumor tissue |
| 8 | Male | 56 | Newly diagnosed | Tumor tissue |
| 9 | Male | 46 | Newly diagnosed | Tumor tissue |
| 10 | Male | 55 | Newly diagnosed | Tumor tissue |
| 11 | Male | 66 | Newly diagnosed | Tumor tissue |
| 12 | Male | 71 | Newly diagnosed | Tumor tissue |
| 13 | Female | 36 | Newly diagnosed | Tumor tissue |
| 14 | Male | 47 | Newly diagnosed | Tumor tissue |
| 15 | Male | 42 | Newly diagnosed | Tumor tissue |
| 16 | Female | 34 | Newly diagnosed | Tumor tissue |
| 17 | Male | 72 | Newly diagnosed | Tumor tissue |
| 18 | Female | 62 | Newly diagnosed | Tumor tissue |
| 19 | Female | 50 | Newly diagnosed | Tumor tissue |
| 20 | Female | 72 | Newly diagnosed | Tumor tissue |
| 21 | Male | 62 | Newly diagnosed | Tumor tissue |
| 22 | Male | 48 | Newly diagnosed | Tumor tissue |
| 23 | Female | 76 | Newly diagnosed | Tumor tissue |
