# Supplementary figures and images for: Cell softness regulates tumorigenicity and stemness of cancer cells
Source: EMBO J. 2020 Dec 4;40(2):e106123. doi: 10.15252/embj.2020106123 (PMC7809788; doi:10.15252/embj.2020106123)

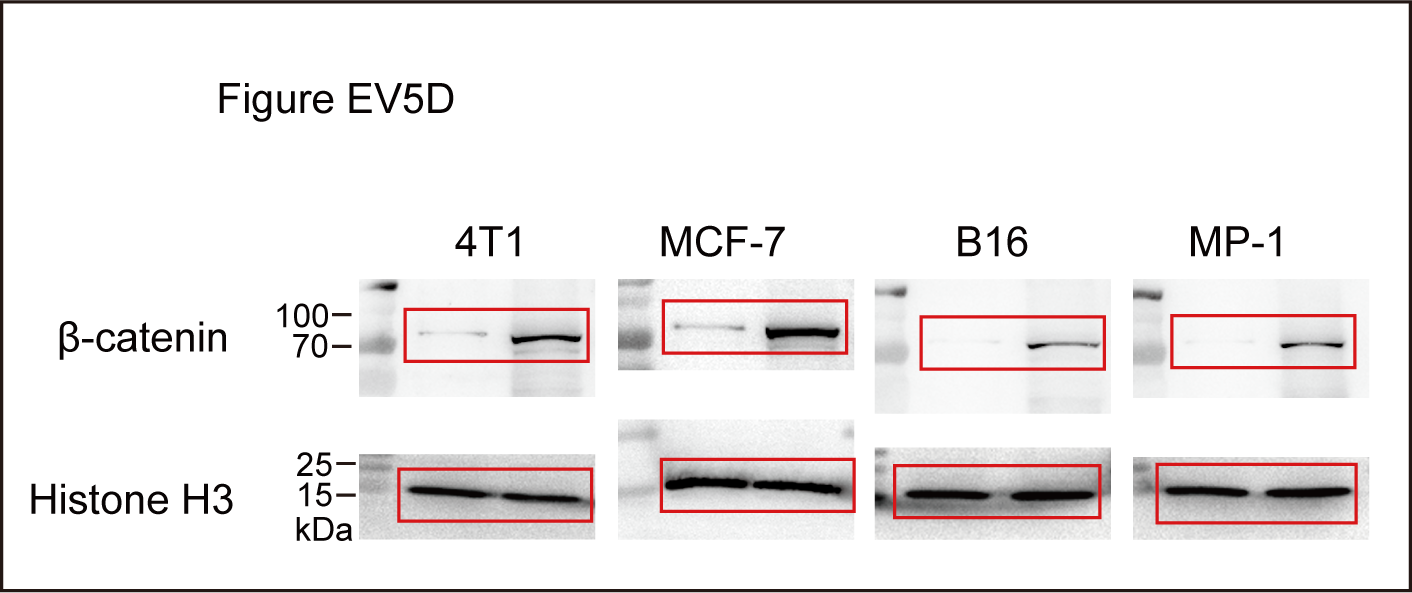

Supplement: Supplementary file 6 — Source Data for Expanded View [file EMBJ-40-e106123-s007.zip › Source_data_for_Figure_EV5.tiff]

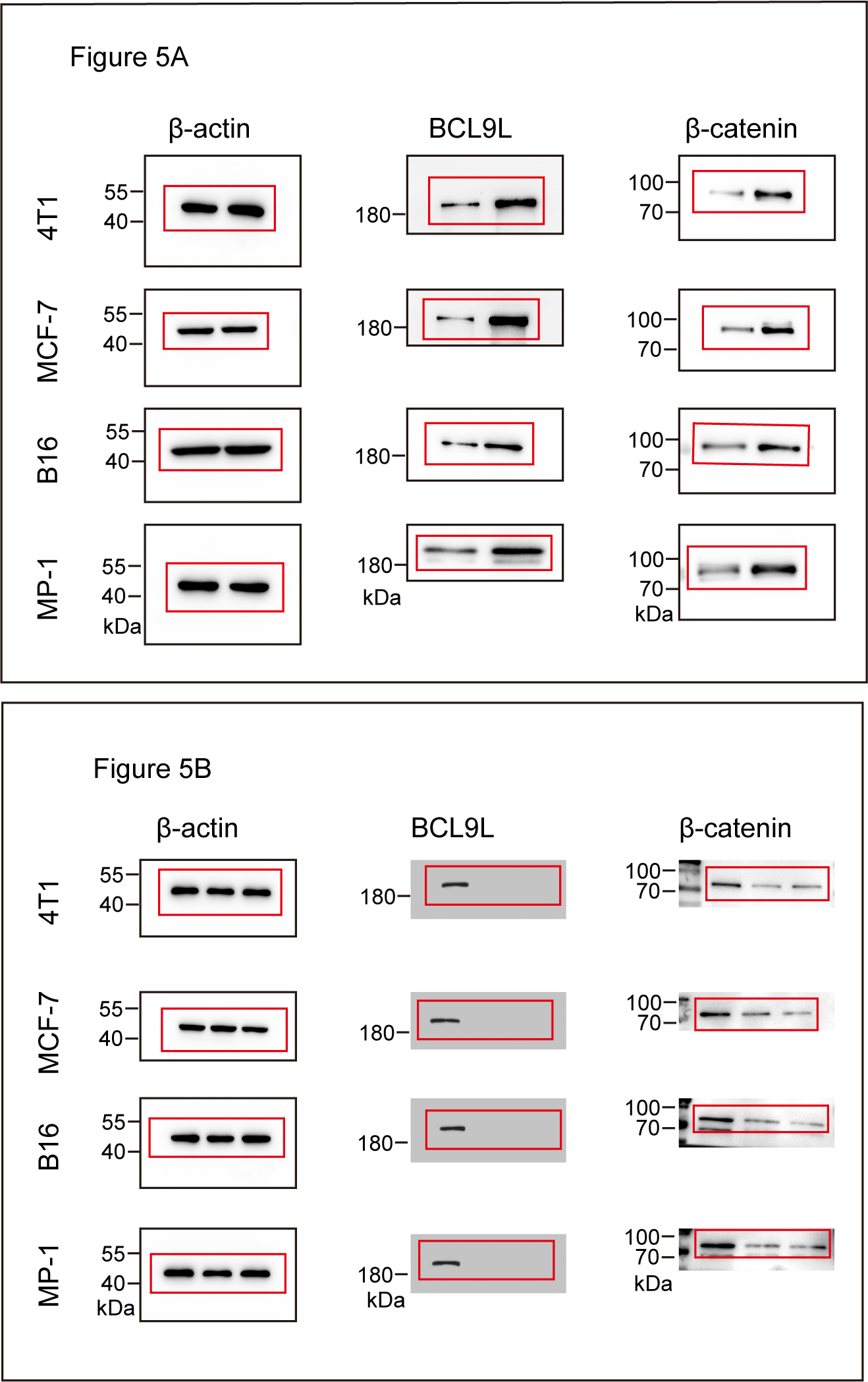

Supplement: Supplementary file 8 — Source Data for Figure 5 [file EMBJ-40-e106123-s006.tif]
